# Cdk1-Mediated Phosphorylation of Human ATF7 at Thr-51 and Thr-53 Promotes Cell-Cycle Progression into M Phase

**DOI:** 10.1371/journal.pone.0116048

**Published:** 2014-12-29

**Authors:** Hitomi Hasegawa, Kenichi Ishibashi, Shoichi Kubota, Chihiro Yamaguchi, Ryuzaburo Yuki, Haruna Nakajo, Richard Eckner, Noritaka Yamaguchi, Kazunari K. Yokoyama, Naoto Yamaguchi

**Affiliations:** 1 Department of Molecular Cell Biology, Graduate School of Pharmaceutical Sciences, Chiba University, Chiba, Japan; 2 Department of Biochemistry and Molecular Biology, Rutgers New Jersey Medical School, Rutgers, The State University of New Jersey, Newark, New Jersey, United States of America; 3 Graduate Institute of Medicine, Kaohsiung Medical University, Kaohsiung, Taiwan; University of Tokyo, Japan

## Abstract

Activating transcription factor 2 (ATF2) and its homolog ATF7 are phosphorylated at Thr-69/Thr-71 and at Thr-51/Thr-53, respectively, by stress-activated MAPKs regulating their transcriptional functions in G1 and S phases. However, little is known about the role of ATF2 and ATF7 in G2/M phase. Here, we show that Cdk1-cyclin B1 phosphorylates ATF2 at Thr-69/Thr-71 and ATF7 at Thr-51/Thr-53 from early prophase to anaphase in the absence of any stress stimulation. Knockdown of ATF2 or ATF7 decreases the rate of cell proliferation and the number of cells in M-phase. In particular, the knockdown of ATF7 severely inhibits cell proliferation and G2/M progression. The inducible expression of a mitotically nonphosphorylatable version of ATF7 inhibits G2/M progression despite the presence of endogenous ATF7. We also show that mitotic phosphorylation of ATF7 promotes the activation of Aurora kinases, which are key enzymes for early mitotic events. These results suggest that the Cdk1-mediated phosphorylation of ATF7 facilitates G2/M progression, at least in part, by enabling Aurora signaling.

## Introduction

The activating transcription factors (ATFs) belong to the AP-1 family of transcription factors [1]. ATF consists of seven members, ATF1∼7. Among these, ATF2 and ATF7 (originally called ATFa) have highly homologous sequences [2]–[4] and are ubiquitously expressed in various tissues [5], [6]. Knockout mutations of ATF2 and ATF7 lead to early postnatal lethality and abnormal behavioral traits reminiscent of isolation-reared wild-type mice, respectively [7], [8]. In addition, the ATF2 and ATF7 double knockout mice die during embryogenesis with abnormalities in the developing liver and heart [2].

ATF2 is mainly controlled by stress-activated protein kinases or protein kinase C (PKC). Jun NH_2_-terminal protein kinase (JNK), p38, and Erk that are activated by stress stimuli can phosphorylate ATF2 at Thr-69 and Thr-71, leading to its transcriptional activation [9]–[14]. Moreover, the phosphorylation of ATF2 at Ser-121 by several PKC isoforms plays a role in the c-Jun-mediated activation of transcription in response to 12-O-tetradecanoylphorbol-13-acetate [15]. It is known that ATF7 is phosphorylated by p38 at Thr-51 and Thr-53, which correspond to Thr-69 and Thr-71 in ATF2, also leading to its transcriptional activation [16], [17]. In contrast to its transcriptional functions, ATF2 has some functions that are independent of transcriptional activation [18]. ATF2 is phosphorylated at Thr-52 by PKCε, which negatively regulates the outer-membrane permeability of mitochondria and inhibits apoptosis during genotoxic stress [19]. In the DNA damage response, the ATM (ataxia-telangiectasia-mutated) protein phosphorylates ATF2 at Ser-490 and Ser-498 to stimulate DNA repair [20], [21]. Thus, ATF2 and ATF7 play important roles in G1 and S phases. However, it is largely unknown whether ATF2 and ATF7 play any role in G2 and M phases.

In this study, we have investigated whether ATF2 and ATF7 are phosphorylated in G2 and M phases in HeLa cells. We show that ATF2 (at Thr-69/Thr-71) and ATF7 (at Thr-51/Thr-53) are phosphorylated by cyclin-dependent kinase 1 (Cdk1) in M phase. Notably, we find that, similar to knockdown of ATF7, the expression of a mitotically nonphosphorylatable ATF7 mutant protein inhibits entry of cells into M phase. Our results suggest that phosphorylation of ATF7 at Thr-51/Thr-53 in M phase is required for G2/M progression, in part by activating Aurora kinases.

## Materials and Methods

### Plasmids

To construct green fluorescent protein (GFP)-tagged ATF2-wt (wild-type) (GFP-ATF2-wt) and GFP-ATF2-TA (T69A/T71A), human ATF2-wt and human ATF2-TA prepared from pcDNA3/FLAG-ATF2-wt and pcDNA3/FLAG-ATF2-TA [15] were subcloned into the pEGFP/C1 vector (Clontech). The pcDNA4/TO/puro vector was generated from the pcDNA4/TO vector (Invitrogen) by replacing the Zeocin-resistant gene with the puromycin-resistant gene of the pPUR vector (BD Biosciences Clontech). pcDNA4/TO/puro/ATF7 (ATF7-wt) was constructed as follows: the HindIII-XhoI fragment of pCR4-TOPO-human ATF7 (Open Biosystems) was subcloned into the HindIII-XhoI site of the pcDNA4/TO/puro vector. The Thr→Ala mutation at positions 51 and 53 (T51A/T53A) (ATF7-TA) in human ATF7 was created by PCR using pcDNA4/TO/puro/ATF7 as a template and the sense primer 5′-TCATTGCAGATCAAGCGCCGGCTCCAACTAGATTCCTGAAGAACTGTGAG-3′ and the antisense primer 5′- CAGGAATCTAGTTGGAGCCGGCGCTTGATCTGCAATGATGACTGAGTCAG-3′. Cyclin B1(R42A)-GFP, a GFP-tagged nondegradable human cyclin B1 mutant, subcloned into the pCMX vector was provided by J. Pines [22]. FLAG-tagged human Cdc2 [Flag-Cdc2(AF)], in which Thr-14 and Tyr-15 residues are mutated, respectively, to Ala and Phe in the pUHD-P1 vector, was provided by R.Y.C. Poon [23], [24].

### Chemicals

The following chemicals were used: thymidine (Sigma), RO-3306 (Cdk1 inhibitor; Calbiochem), SB202190 (p38 inhibitor; Calbiochem), SP600125 (JNK inhibitor; Biomol international), U0126 (MEK inhibitor; Calbiochem), Gö6976 (PKC inhibitor; Calbiochem), MG132 (proteasome inhibitor; Peptide Institute, Inc.), ZM 447439 (Aurora B inhibitor; JS Research Chemicals Trading), and MLN 8237 (Aurora A inhibitor; Selleck Chemicals), and monastrol (kinesin inhibitor; Enzo Life Sciences).

### Antibodies

The following antibodies were used: phospho-ATF2[pT71] (which recognizes both pATF2 and pATF7) (#9221; Cell Signaling Technology), ATF2[N-96] (which recognizes both ATF2 and ATF7) (sc-6233; Santa Cruz Biotechnology, Inc.), ATF2[SS-16] (specific for ATF2) (A4086; Sigma-Aldrich), ATF7 (specific for ATF7) (SAB2500131 and HPA003384; Sigma-Aldrich), cyclin A (clone CY-A1, C4710; Sigma-Aldrich), cyclin B1 (#4135; Cell Signaling Technology), phospho-CDK1[Y-15] (clone 10A11) (#4539; Cell Signaling Technology), CDK1[p34] (sc-574; Santa Cruz Biotechnology, Inc.), phospho-Aurora A/B/C (clone D13A11; Cell Signaling Technology), Aurora A (IAK1, clone 4; BD Bioscience), Aurora B (AIM-1, clone 6; BD Bioscience), phospho-histone H3 Ser-10 (clone 6G3, #9706S; Cell Signaling Technology), α-tubulin (clone MCA78G; Serotec), and actin (clone C4, MAB1501; Millipore). Horseradish peroxidase (HRP)-F(ab′)_2_ secondary antibodies were purchased from Amersham Biosciences. Alexa Fluor 488-, Alexa Fluor 546-, and Alexa Fluor 647-labeled IgG secondary antibodies were purchased from Invitrogen.

### Cells and transfection

HeLa S3 cells (Japanese Collection of Research Bioresources, Osaka, Japan) and tetracycline repressor (TR)-expressing HeLa S3 cells (HeLa S3/TR, clone A3f5) [25], [26] were cultured in Iscove’s modified Dulbecco’s medium (IMDM) containing 1% fetal bovine serum (FBS) and 4% bovine serum. Cells seeded in a 35-mm culture dish were transiently transfected with 1 µg of plasmid vector using 5 µg of linear polyethylenimine (25 kDa; Polyscience, Inc., Warrington, PA, USA) [27]. To generate stable cell lines for tetracycline-inducible ATF7-wt or ATF7-TA expression, HeLa S3/TR cells were stably transfected with pcDNA4/TO/puro/ATF7-wt or pcDNA4/TO/puro/ATF7-TA, and cell clones expressing inducible ATF7-wt or ATF7-TA were selected in 350 ng/ml puromycin. Expression of ATF7-wt or ATF7-TA was induced by addition of 1 µg/ml Dox, a tetracycline derivative.

### RNA interference

Knockdown of human ATF7 was performed using shRNAs for silencing ATF7 [5′-GCTAGATTTGATGACATATTA-3′, which is a sequence in the 3′UTR (Sigma MISSION shRNA library), and 5′-GTCACATTACTACGCAATG-3′, which is a sequence in the CDS] (S1A Fig.). Knockdown of human ATF2 was performed with an shRNA for silencing ATF2 (5′-GAAGAAGTGGGTTTGTTTA-3′, which is a common sequence among all 13 transcript variants) [28], [29]. The oligonucleotides used for shRNA were annealed and subcloned into the XbaI and BglII sites of the pENTR4-H1 vector (provided by Hiroyuki Miyoshi). The EBNA1-based episomal pEBMulti-H1 vector, which encodes the H1 promoter and a neomycin-resistant gene, was generated from the pEBMulti vector (Wako Pure Chemical Industries, Osaka, Japan) by replacing the CAG promoter with the H1 promoter. The oligonucleotides used for shRNA were annealed and subcloned into the pEBMulti-H1 vector. To generate ATF2 or ATF7 knockdown cells, HeLa S3/TR cells or HeLa S3/TR expressing inducible ATF7-wt or ATF7-TA were transfected with pEBMulti-neo/shATF2 or pEBMulti-neo/shATF7, and selected in 600 µg/ml G418. Viable parental HeLa S3/TR cells were not detected after a 5-day selection using 600 µg/ml G418.

### Flow cytometry

For cell-cycle analysis, cells detached by trypsinization were fixed in 4% paraformaldehyde for 1 h, and permeabilized with 70% ethanol for at least 1 h at −30°C [26], [30]. Fixed cells were permeabilized and blocked in phosphate-buffered saline (PBS) containing 0.1% saponin and 3% bovine serum albumin (BSA) for 30 min at room temperature. After washing with PBS containing 0.1% Tween 20, cells were reacted with anti-phospho-histone H3 Ser-10 antibody for 1 h at room temperature, then stained with AF647-conjugated anti-mouse IgG antibody for 1 h. Subsequently, cells were treated with 200 µg/ml RNase A and 50 µg/ml propidium iodide (PI) at 37°C for 30 min to stain DNA. A minimum of 5,000 cells per sample was analyzed by flow cytometry using a Guava easyCyte (Millipore) equipped with a 488-nm blue laser and a 640-nm red laser using liner amplification. Data acquired with a Guava easyCyte apparatus were analyzed using Flowing Software version 2.5.0 (Perttu Terho, Centre for Biotechnology, Turku, Finland). Cell debris was excluded by gating on forward scatter and pulse-width profiles.

### Cell synchronization

The HeLa S3 cell line is a HeLa cell variant that can be highly synchronized at a various stages of the cell cycle [26], [27], [31]–[33]. HeLa S3 cells were treated with 4 mM thymidine for 24 h, washed and released into thymidine-free fresh medium. After 11 h of release from S-phase arrest, mitotic cells were collected by mitotic shake-off. Cells were grown for 24 h in medium with 4 mM thymidine, an additional 9 h without thymidine, then an additional 15 h with 4 mM thymidine (DTB), and released into fresh medium for 10 h (G2 phase) and 11∼12 h (M phase). Alternatively, to synchronize exponentially growing cells at M phase, cells were treated with 4 mM thymidine for 24 h. After washing with PBS, cells were released into thymidine-free medium and cultured for 5 h. The cells were then incubated with 9 µM RO-3306 for 10 h, to arrest cells at late G2 phase. The G2-arrested cells were washed with PBS supplemented with Ca^2+^ and Mg^2+^ and released into prewarmed fresh medium [26]. Cell-cycle distribution was determined by flow cytometry.

### Immunofluorescence

Immunofluorescence staining was performed as described previously [34]–[36]. In brief, HeLa S3 cells were cultured in IMDM containing 1% FBS and 4% bovine serum at 37°C. Cells were fixed in 4% paraformaldehyde for 15 min at room temperature. Fixed cells were permeabilized with 100% methanol for 10 min at −20°C and blocked in PBS containing 0.1% saponin and 3% BSA for 30 min at room temperature. Cells were stained with a primary and a secondary antibody for 1 h each. For DNA staining, cells were subsequently treated with 200 µg/ml RNase A for 30 min and 20 µg/ml PI for 30 min. Stained cells were mounted with an antifade reagent. Confocal and Nomarski differential-interference contrast images were obtained using an FV500 laser scanning microscope (Olympus). To ensure that there was no bleed through from the Alexa Fluor 488 signal into the red channel, Alexa Fluor 488 and Alexa Fluor 546 were independently excited at 488 nm and 543 nm, respectively. Emission signals were detected between 505 and 525 nm for Alexa Fluor 488 and between 560 and 600 nm for Alexa Fluor 543. Composite figures were prepared using Photoshop CS6 and Illustrator CS6 (Adobe).

### Western blotting and immunoprecipitation

Cell lysates were prepared in SDS-PAGE sample buffer or Triton X-100 lysis buffer (10 mM HEPES, pH 7.8, 5% glycerol, 1% Triton X-100, 5 mM EDTA, 50 mM NaF, 20 mM β-glycerophosphate, 50 µg/ml aprotinin, 100 µM leupeptin, 25 µM pepstatin, 10 mM Na_3_VO_4_, and 1 mM PMSF), and subjected to SDS-PAGE and electrotransferred onto a polyvinylidene difluoride membrane (Millipore). Prestained XL-Ladder Broad (Apro Science, Japan) was used for molecular size markers. Immunodetection was performed by ECL (Amersham Biosciences), as described previously [24], [25], [31], [37]–[40]. Sequential reprobing of membranes with a variety of antibodies was performed after inactivation of HRP by 0.1% NaN_3_ or stripping buffer, unless otherwise stated. Results were analyzed using a ChemiDoc XRSPlus analyzer (Bio-Rad). Immunoprecipitation was performed using antibody-precoated protein-G beads, as described previously [37], [39], [40].

## Results

### Involvement of ATF2 and ATF7 in cell-cycle progression

**Figure 1:**
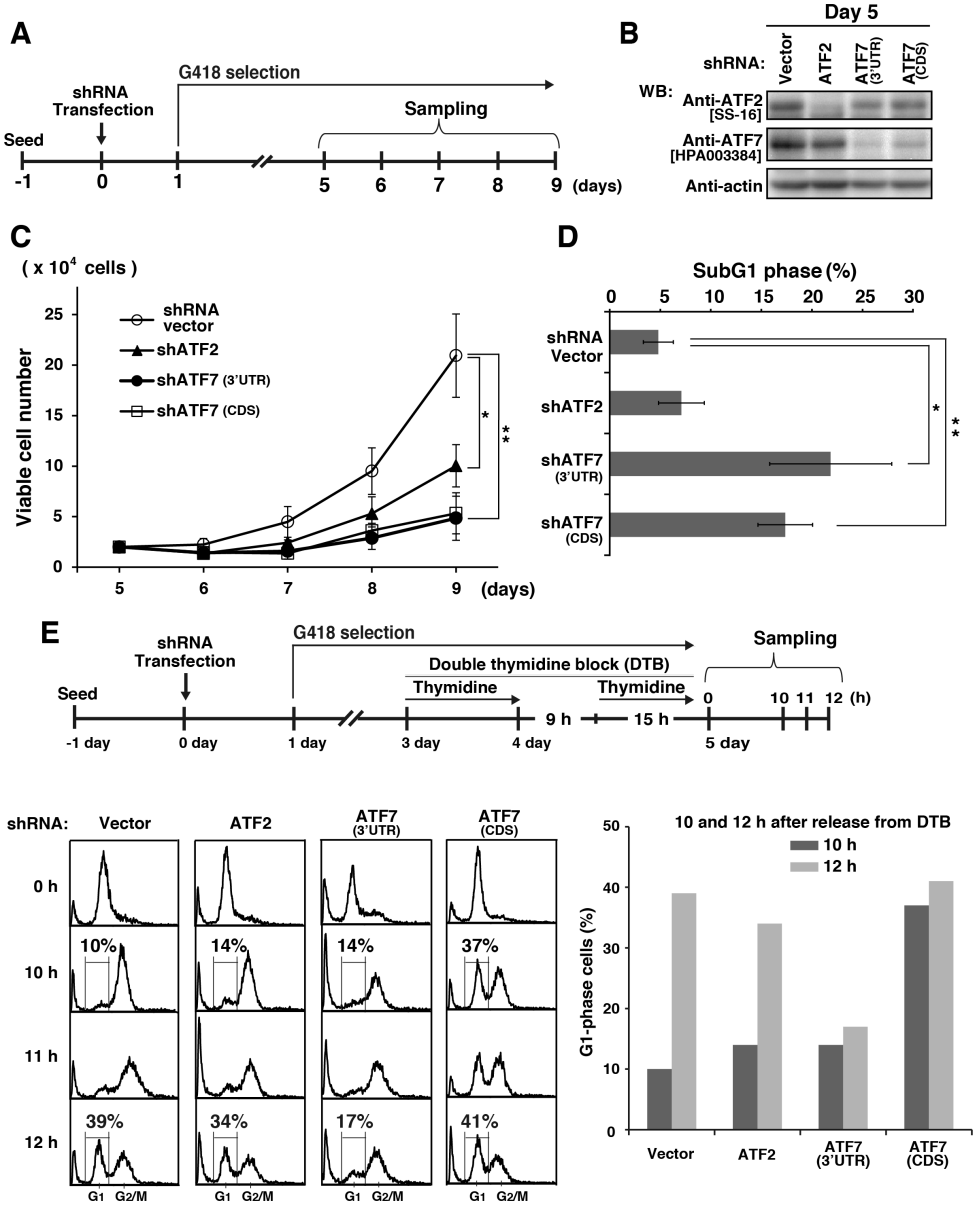
Effect of ATF2 or ATF7 knockdown on cell-cycle progression. (**A**) Schematic depiction of our knockdown method. Cells were transfected with the episomal pEBMulti vector for shRNA expression and selected in 600 µg/ml G418. (**B**) Cells were transfected with control vector, ATF2 shRNA, ATF7 shRNA(3′UTR), or ATF7 shRNA(CDS) and collected on day 5. Whole cell lysates were analyzed by Western blotting (WB) using anti-ATF2[SS-16], anti-ATF7[HPA003384], and anti-actin (loading control) antibodies. Full-length blots are presented in S7A Fig. (**C**) The number of cells was counted on days 6∼9 after shRNA transfection. Values are means ± standard deviation (SD), n = 3 independent experiments (only shATF7(CDS), n = 2 independent experiments). Asterisks indicate the significant differences (*P<0.05; **P<0.01), as calculated by Student’s *t*-test. (**D**) Cells transfected with control vector, ATF2 shRNA, ATF7 shRNA(3′UTR), or ATF7 shRNA(CDS) were collected on day 7 and stained with propidium iodide (PI). SubG1 cells were quantitated by flow cytometry. Values are means ± SD, n  = 3 independent experiments. Asterisks indicate the significant differences (*P<0.05; **P<0.01), as calculated by Student’s *t*-test. (**E**) Schematic depiction of our synchronization method. Cells transfected with control vector, ATF2 shRNA, ATF7 shRNA(3′UTR), or ATF7 shRNA(CDS) were synchronized using double thymidine block (DTB) in the presence of 600 µg/ml G418 and released into thymidine-free medium for 10∼12 h. Cells were stained with PI for analyzing cell cycle progression by flow cytometry (left panels). The percentages of G1-phase cells were compared between 10 h and 12 h after release from DTB (right graph).

The human ATF2 and human ATF7 genes generate 13 and six transcript variants, respectively. To examine whether ATF2 and ATF7 are involved in cell-cycle progression into G2/M phases, we chose the following knockdown target sequences: the first sequence was common among the human ATF2 variants, and the other two sequences were the sequences in the coding sequence (CDS) and the 3′-untranslated region (3′UTR) of human ATF7 (see Materials and Methods, S1A Fig.). We tried to knock down endogenous ATF2 and ATF7 in HeLa S3 cells using short hairpin RNAs (shRNAs). After transfection of cells with an EBNA1-based episomal vector, which encoded shRNA against ATF2 (shATF2) or ATF7 [shATF7(CDS) or shATF7(3′UTR)], we selected knockdown cells in G418-containing medium. Western blot analysis showed that the level of endogenous ATF2 or ATF7 was significantly decreased 5 days post transfection (Fig. 1A–B S1B–E Fig.). The use of shATF7(CDS) or shATF7(3′UTR) resulted in efficient knockdown of ATF7 proteins. Intriguingly, knockdown of ATF2 or ATF7 inhibited the rate of cell proliferation and decreased the number of M-phase cells (Fig. 1C, S1F Fig.). Note that ATF7 knockdown had severe inhibitory effects on cell proliferation compared with the ATF2 knockdown (Fig. 1C, S1F Fig.). These results suggest that ATF2 and ATF7, in particular ATF7, play a role in cell proliferation, in addition to their well-established role during stress and DNA-damage responses in G1/S phases. Moreover, ATF7 knockdown also strongly affected the level of the ATF2 protein (Fig. 1B, S1B-E Fig.), although none of the ATF2 transcript variants contain any detectable homology to the shATF7(3′UTR) and shATF7(CDS) sequences. These findings suggest that the level of the ATF2 protein is dependent on that of ATF7.

Next, we examined by flow cytometry whether knockdown of ATF2 and ATF7 affected cell viability. We found that the number of cells in subG1 phase was significantly increased upon ATF7 knockdown 7 days post transfection (Fig. 1D). To examine further whether ATF2 and ATF7 play a role in cell-cycle progression, we highly synchronized knockdown cells using a double-thymidine block (DTB) method. After transfection with either vector, shATF2 or shATF7(3′UTR), cells that were synchronized in G1/S phase appeared to progress normally into G2/M phase 10 h after release from DTB. It is of interest to note that 12 h after release from DTB, knockdown of ATF7 by shATF7(3′UTR) severely decreased the number of G1-phase cells compared with knockdown of ATF2 by shATF2 (Fig. 1E), which indicates that ATF7 knockdown by shATF7(3′UTR) drastically inhibits the progression of cells from M phase to G1 phase. Moreover, knockdown of ATF7 by shATF7(CDS) appeared to partially arrest cells at G1 phase (Fig. 1E, S2 Fig.), in addition to inhibiting the progression from M phase to G1 phase (Fig. 1E, compare 10 h with 12 h), indicating that the ATF7 transcript variant 6 (long noncoding RNA) (S1 Fig.) is involved in the progression from G1 phase to S phase. Taken together, these results suggest that ATF7 knockdown primarily affects cell-cycle progression during M phase.

### Phosphorylation of ATF2 at Thr-69/Thr-71 and of ATF7 at Thr-51/Thr-53 in M phase

**Figure 2:**
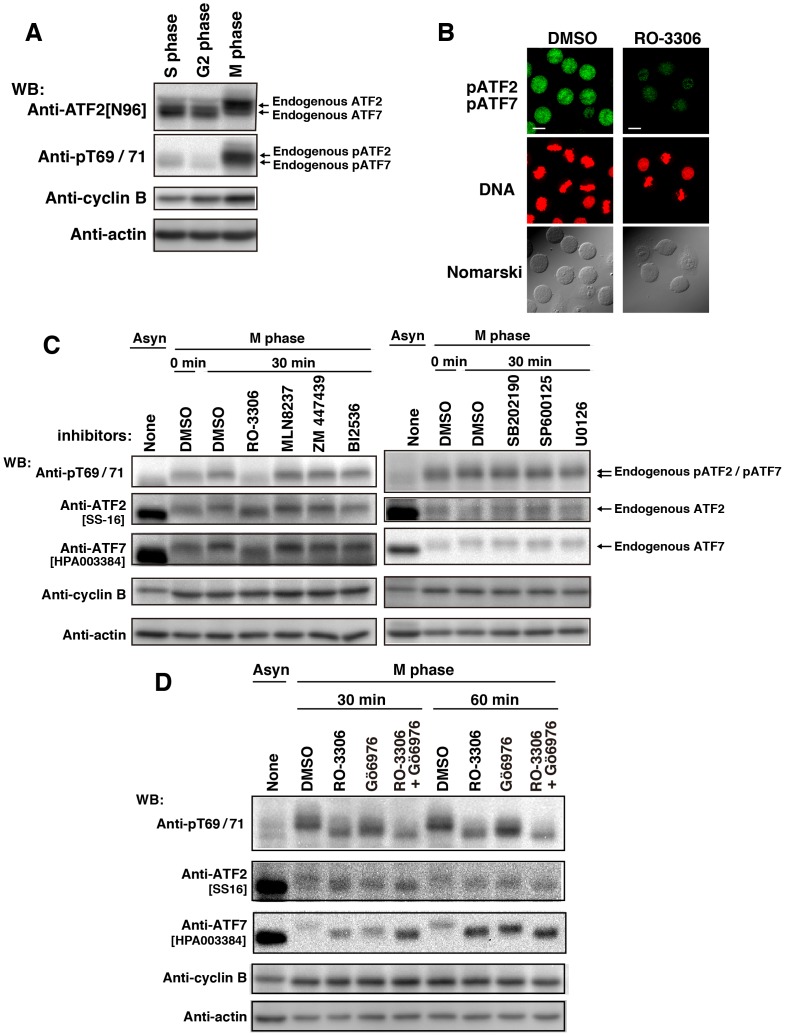
Mitotic phosphorylation of ATF2 and ATF7 by Cdk1. (**A**) Cells were synchronized at S, G2, or M phases. Mitotic cells were collected by mitotic shake-off, and whole cell lysates were analyzed by WB using antibodies against ATF2/ATF7 (ATF2[N96]), pATF2/pATF7 (pT69/71), cyclin B1, and actin. (**B–D** Cells were arrested at G2 phase using 9 µM RO-3306 and released into RO-3306-free medium containing 10 µM MG132. (**B**) At 20 min after release, cells were treated for an additional 60 min in the presence of 10 µM MG132 together with DMSO (solvent control) or 9 µM RO-3306. Cells were doubly stained with anti-pT69/71 antibody and PI. Cells stained with anti-histone pT69/71 antibody are pseudo-colored as green. Scale bars, 20 µm. (**C**) At 20 min after release from RO-3306, cells were treated with DMSO (solvent control), 9 µM RO-3306, 1 µM MLN8237, 10 µM ZM447439, 1 µM BI2536, 20 µM SB202190, 20 µM SP600125, or 20 µM U0126 for a further 30 min in the presence of 10 µM MG132. Whole cell lysates were analyzed by WB. Asyn, asynchronous. (**D**) At 20 min after release, cells were treated for an additional 30 or 60 min in the presence of 10 µM MG132 together with DMSO (solvent control), 9 µM RO-3306, 10 µM Gö6976, or RO-3306 plus Gö6976. Whole cell lysates were analyzed by WB. Asyn, asynchronous. Full-length blots are presented in S7B, S8A–B and S9 Figs.

ATF2 and ATF7 are activated by threonine phosphorylation [12], [16]. The amino acid sequence surrounding the threonine-phosphorylation sites of ATF2 is identical to that of ATF7. The use of anti-phosphorylated ATF2[pT71] antibody (anti-pT69/71) enabled us to detect the phosphorylation of Thr-69/Thr-71 of ATF2 and Thr-51/Thr-53 of ATF7. In some experiments anti-ATF2[N96] antibody, which recognizes both ATF2 and ATF7 [41], was used. To examine the phosphorylation states of endogenous ATF2 at Thr-69/Thr-71 and endogenous ATF7 at Thr-51/Thr-53 in the cell cycle, we synchronized cells at S, G2, and M phases, and used anti-pT69/71 and anti-ATF2[N96] antibodies. Western blotting with anti-pT69/71 antibody revealed that endogenous ATF2 and ATF7 were strongly phosphorylated in M phase. Strikingly, the appearance of ATF2 and ATF7 protein forms with lower mobility on SDS-PAGE, suggestive of phosphorylation, was only found in M phase (Fig. 2A). To identify the kinase that phosphorylates ATF2 and ATF7 in M phase, we used inhibitors of various mitotic kinases, such as Cdk1, Aurora A, Aurora B, and Polo-like kinase 1. We also used anti-ATF2 antibody[SS-16] and anti-ATF7 antibody[HPA003384], which recognize specific sequences of ATF2 and ATF7, respectively. Notably, the phosphorylation levels of ATF2 and ATF7 in M phase were drastically decreased in cells treated with RO-3306, an inhibitor of Cdk1 (Fig. 2B, C). In addition, we noticed that the amino acid sequence surrounding the phosphorylation sites of ATF2 and ATF7 complied with the Cdk1 phosphorylation consensus sequence (S/T*-P-x-K/R). Since MAP kinases and PKC phosphorylate ATF2 and ATF7 in G1 phase [12], [15], [16], we examined whether MAP kinases and PKC were capable of phosphorylating ATF2 at Thr-69/Thr-71 and ATF7 at Thr-51/Thr-53 in M phase. However, treatment with inhibitors of MAP kinases such as p38, JNK, and Erk did not decrease the phosphorylation levels of ATF2 at Thr-69/Thr-71 and ATF7 at Thr-51/Thr-53 in M phase (Fig. 2C). Moreover, inhibition of PKC by Gö6976 did not affect the phosphorylation levels of ATF2 at Thr-69/Thr-71 and of ATF7 at Thr-51/Thr-53, although PKC inhibition partially reduced the mobility shift of ATF2 and ATF7 (Fig. 2D). These results suggest that Cdk1, but not MAP kinases or PKC, phosphorylates ATF2 at Thr-69/Thr-71 and ATF7 at Thr-51/Thr-53 in M phase.

**Figure 3:**
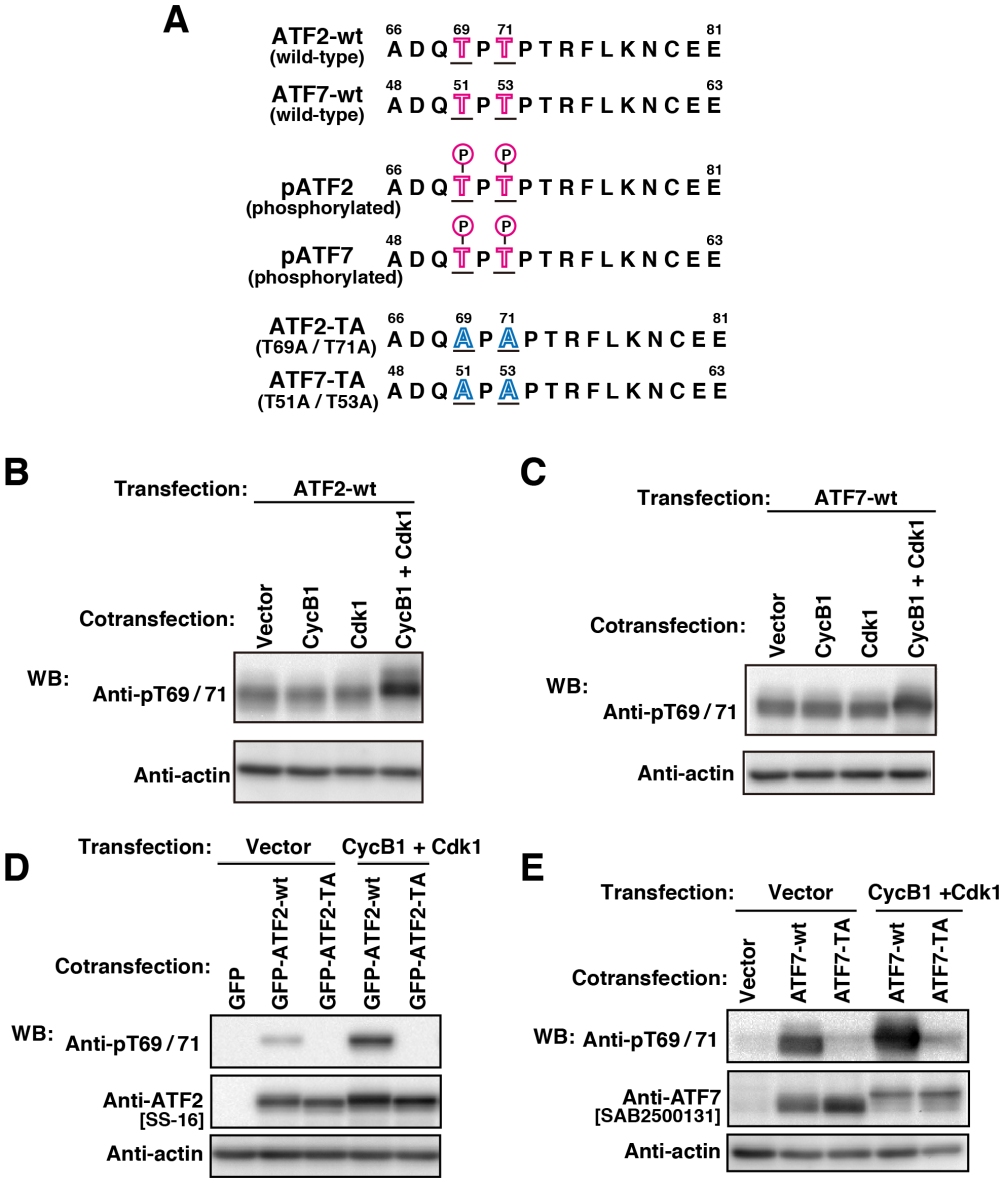
Phosphorylation of ATF2 (at Thr69/Thr71) and ATF7 (at Thr51/Thr53) by Cdk1-cyclin B1. (**A**) Amino acid sequence of ATF2 and ATF7 with the common phosphorylation sites. pATF2, phosphorylated ATF2; pATF7, phosphorylated ATF7; ATF2-TA and ATF7-TA, T→A mutants. (**B–E** Cells that were transiently transfected with the indicated constructs were cultured for 24 h, and whole cell lysates were analyzed by WB. (**B**) Cells were cotransfected with ATF2-wt together with control vector, cyclin B1, Cdk1 or cyclin B1 plus Cdk1. (**C**) Cells were cotransfected with ATF7-wt together with control vector, cyclin B1, Cdk1, or cyclin B1 plus Cdk1. (**D**) Cells were cotransfected with control vector together with GFP-ATF2-wt or GFP-ATF2-TA, or cells were cotransfected with cyclin B1 plus Cdk1 together with GFP-ATF2-wt or GFP-ATF2-TA. (**E**) Cells were cotransfected with control vector together with ATF7-wt or ATF7-TA, or cells were cotransfected with cyclin B1 plus Cdk1 together with ATF7-wt or ATF7-TA. Full-length blots are presented in S10 Fig.

To substantiate further the mitotic phosphorylation of ATF2 and ATF7 by Cdk1, we constructed the plasmids encoding ATF2-wt, ATF7-wt, ATF2-TA (Thr-69/Thr-71→Ala-69/Ala-71), and ATF7-TA (Thr-51/Thr-53→Ala-51/Ala-53) (Fig. 3A). Cells were cotransfected with ATF2-wt or ATF7-wt together with cyclin B1, Cdk1, or cyclin B1 plus Cdk1 (Fig. 3B, C). We found that the phosphorylation levels of ATF2-wt and ATF7-wt were increased by cotransfection of cells with cyclin B1 plus Cdk1. However, the phosphorylation levels of ATF2-wt and ATF7-wt were not increased by cotransfection with cyclin B1 or Cdk1 alone (Fig. 3B, C). The phosphorylation site mutant ATF2-TA or ATF7-TA proteins were not phosphorylated when cyclin B1 plus Cdk1 were cotransfected (Fig. 3D, E), indicating that the Cdk1-cyclinB1 complex indeed phosphorylates ATF2 at Thr-69/Thr-71 and ATF7 at Thr-51/Thr-53 Cdk1 consensus motives.

### Phosphorylation of ATF2 and ATF7 from prophase to anaphase

**Figure 4:**
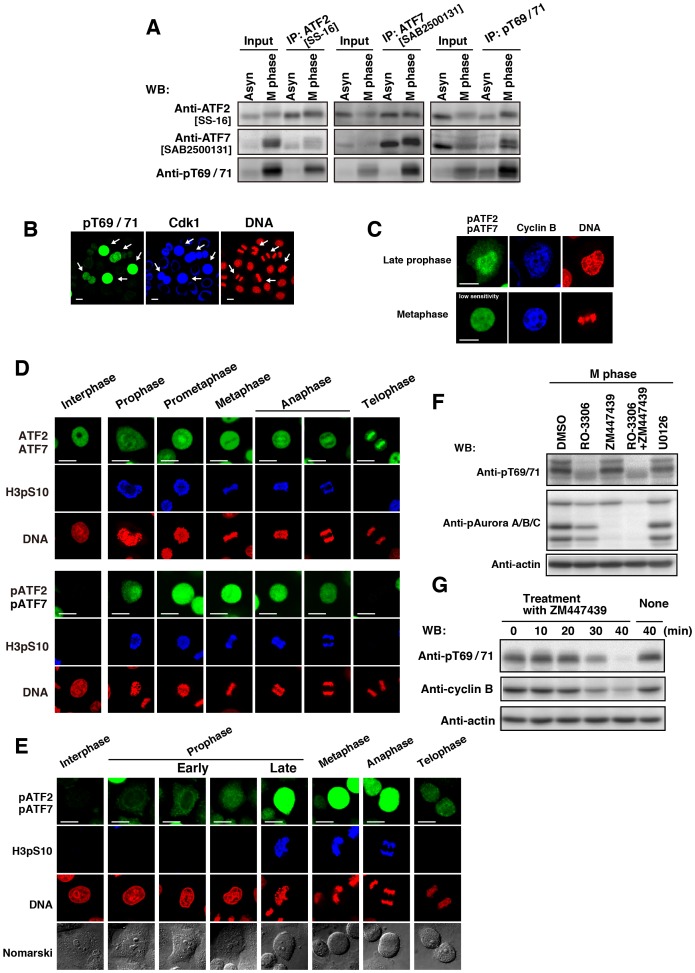
Phosphorylation of ATF2 and ATF7 from prophase to anaphase. (**A–E** Cells were synchronized using single-thymidine block and released into thymidine-free medium for 11 h. (**A**) Mitotic cells collected by mitotic shake-off and asynchronous (Asyn) cells were lysed with Triton X-100. Endogenous ATF2, ATF7, and pATF2/pATF7 were individually immunoprecipitated from Triton X-100 cell lysates using antibodies specific for ATF2 [SS-16], ATF7 [SAB2500131], and pATF2/pATF7 (pT69/71). Full-length blots are presented in S11A Fig. The gels for ATF7 IP blotted with anti-pT69/71 antibody and anti-ATF2 and anti-ATF7 antibodies have been run under the same experimental conditions. (**B**) Cells were triply stained with anti-pT69/71 and anti-Cdk1 antibodies and PI (for DNA). Anti-Cdk1-stained cells are pseudo-colored as blue. Scale bars, 20 µm. Arrows indicate mitotic cells. (**C**) Cells were triply stained with anti-pT69/71 and anti-cyclin B1 antibodies and PI (for DNA). Staining of pATF2/pATF7 was recorded with a low sensitivity. Anti-cyclin B1-stained cells are pseudo-colored as blue. Scale bars, 20 µm. (**D**) Cells were triply stained with anti-ATF2[N96] (upper panels) or anti-pT69/71 (lower panels) antibody, anti-histone H3pS10 antibody (for M phase) and PI (for DNA). Scale bars, 20 µm. (**E**) Cells were triply stained with anti-pT69/71 antibody, anti-histone H3pS10 antibody (for M phase), and PI (for DNA). Scale bars, 20 µm. (**F**) Cells were arrested at G2 phase using 9 µM RO-3306 and were released into RO-3306-free medium containing 10 µM MG132. At 20 min after release, cells were treated for an additional 60 min in the presence of 10 µM MG132 together with DMSO, 9 µM RO-3306, 10 µM ZM447439, RO-3306 plus ZM447439, or 20 µM U0126. Whole cell lysates were analyzed by WB. Full-length blots are presented in S11B Fig. (**G**) Cells arrested at G2 phase by treatment with 9 µM RO-3306 were released into RO-3306-free medium containing 100 µM monastrol for 1 h. The monastrol-arrested cells were collected by mitotic shake off and incubated with 10 µM ZM447439 for the indicated times (induction of mitotic slippage). Whole cell lysates were analyzed by WB. Full-length blots are presented in S11C Fig.

To examine whether ATF2 was able to form a complex with ATF7, we immunoprecipitated endogenous ATF2 and endogenous ATF7 using the specific antibodies ATF2[SS-16] and ATF7[SAB2500131 or HPA003384], respectively. The results of co-immunoprecipitation experiments showed that ATF2 was associated with ATF7, and that ATF7 was associated with ATF2 (Fig. 4A). In addition, phosphorylated ATF2 and ATF7 could be immunoprecipitated using anti-pT69/71 antibody from cell extracts prepared from M phase cells. Next, untransfected cells were co-immunostained for endogenous, phosphorylated ATF2/ATF7 and endogenous Cdk1. Phosphorylated ATF2/ATF7 colocalized with Cdk1 and cyclin B1 in M phase (Fig. 4B, arrows, Fig. 4C). To compare the localization of ATF2/ATF7 and phosphorylated ATF2/ATF7, we used anti-ATF2[N96], anti-pT69/71, and anti-phospho-histone H3 at Ser-10 [H3pS10] antibodies. Anti-ATF2[N96] antibody, which recognizes both ATF2 and ATF7, reacted with the nucleus in interphase, the cytoplasm from prophase to anaphase, and chromosomes in telophase (Fig. 4D, upper panels). Anti-pT69/71 antibody reacted with the cytoplasm from prophase to anaphase (Fig. 4D, lower panels), and the mitotic phosphorylation of ATF2 and ATF7 preceded the onset of the phosphorylation of histone H3 at Ser-10 (Fig. 4E). Anti-H3pS10 and DNA staining confirmed the presence of cells undergoing prophase, metaphase, or anaphase. The use of the Cdk1 inhibitor RO-3306 and the Aurora kinase inhibitor ZM447439 showed that phosphorylation of ATF2 and ATF7 was not inhibited by ZM447439 and was inhibited by RO-3306, a result that further substantiates the notion that the mitotic phosphorylation of ATF2 and ATF7 takes place upstream of the activation of Aurora kinases (Fig. 4F). Moreover, to examine the relationship between the levels of phosphorylated ATF2/ATF7 and cyclin B1, we examined at the metaphase-anaphase transition. Mitotic slippage was induced by ZM447439 after cells were arrested by the spindle checkpoint-inducer monastrol. We observed that phosphorylation of ATF2/ATF7 was decreased to basal levels in anaphase and telophase, where cyclin B1 are low due to active degradation (Fig. 4G). These results suggest that ATF2 and ATF7 are phosphorylated by Cdk1-cyclin B1 from early prophase to anaphase.

### Role of the phosphorylation of ATF7 in G2/M progression

**Figure 5:**
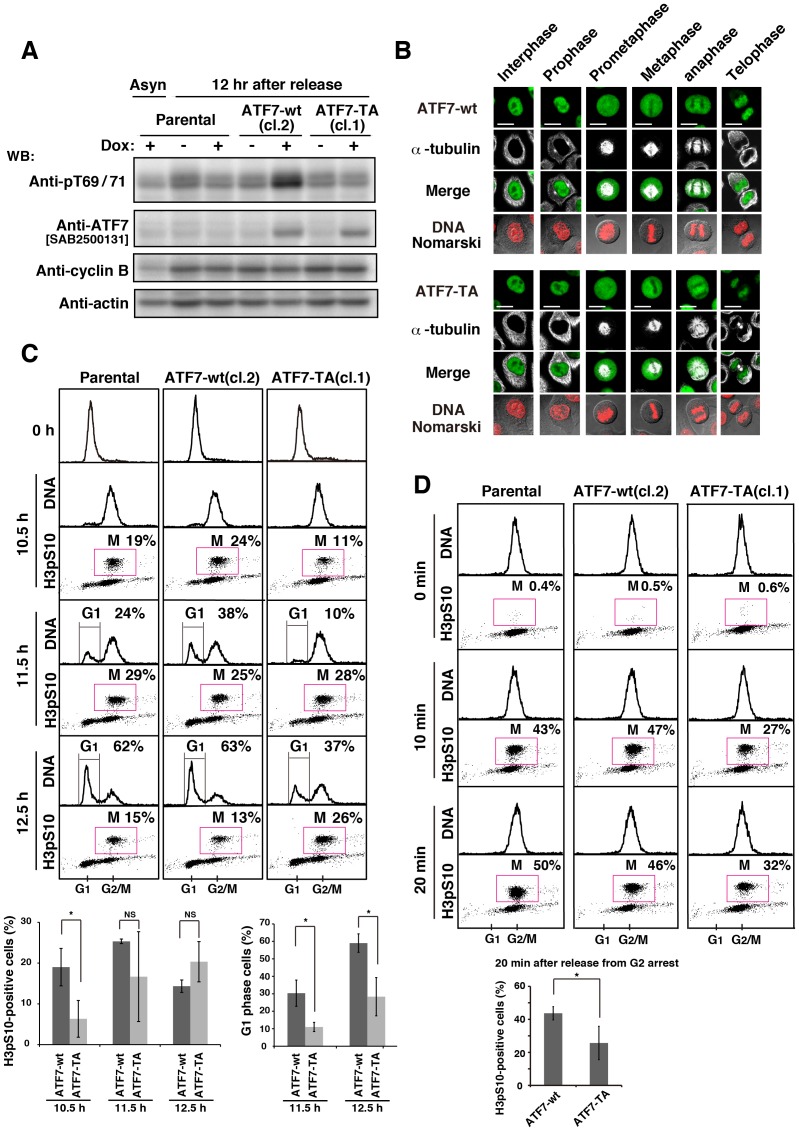
Role of the phosphorylation of ATF7 in G2/M progression. (**A**) Parental HeLa S3/TR, HeLa S3/TR/ATF7-wt (cl.2), or HeLa S3/TR/ATF7-TA (cl.1) cells were synchronized using DTB and released into thymidine-free medium containing 1 µg/ml Dox for 12 h. Whole cell lysates were analyzed by WB. Full-length blots are presented in S12A Fig. (**B**) HeLa S3/TR/ATF7-wt (cl.2) (upper panels) or HeLa S3/TR/ATF7-TA (cl.1) cells (lower panels) were synchronized using single-thymidine block and released into thymidine-free medium containing 1 µg/ml Dox for 11 h. Cells were triply stained with anti-ATF7[SAB2500131] and anti-α-tubulin antibodies and PI (for DNA). Scale bars, 20 µm. (**C, D**) Cells were stained with anti-histone H3pS10 antibody (for M phase) and PI for analyzing cell-cycle progression by flow cytometry. (**C**) Parental HeLa S3/TR, HeLa S3/TR/ATF7-wt (cl.2), or HeLa S3/TR/ATF7-TA (cl.1) cells were synchronized using DTB and released into thymidine-free medium containing 1 µg/ml Dox for 10.5∼12.5 h. Two-dimensional histograms (DNA vs histone H3pS10) are presented together with DNA histograms, and the percentages of cells in G1 and M phases were measured. Cells in M and and G1 phases were quantitated from the results of S4 Fig. Values are means ± SD (three independent clones). Asterisks indicate the significant difference (*P<0.05; NS, not significant), as calculated by Student’s *t*-test. (**D**) Parental HeLa S3/TR, HeLa S3/TR/ATF7-wt (cl.2), or HeLa S3/TR/ATF7-TA (cl.1) cells were cultured in the presence of 9 µM RO-3306 for 10 h and treated with 1 µg/ml Dox for the last 5 h. The cells that were arrested at G2 phase were released into RO-3306-free medium containing 1 µg/ml Dox and incubated for 0, 10, and 20 min. Two-dimensional histograms (DNA vs histone H3pS10) are presented together with DNA histograms, and M-phase cells were quantitated. Values are means ± SD, n  = 3 independent experiments. An asterisk indicates the significant difference (*P<0.05), as calculated by Student’s *t*-test.

Although ATF2 and ATF7 were similarly threonine-phosphorylated in M phase (Fig. 2, 4A), ATF7 knockdown affected cell proliferation and cell-cycle progression more severely compared with ATF2 knockdown (Fig. 1C–E, S1F Fig.). Therefore, we focused on scrutinizing the role of ATF7 in M phase. To examine the role of the mitotic threonine-phosphorylation of ATF7 in cell-cycle progression, we generated three independent, stable HeLa S3/TR cell clones that expressed tetracycline-inducible ATF7-wt or ATF7-TA (see Fig. 3A). Western blot analysis showed that expression of ATF7-wt and ATF7-TA was induced by treatment with doxycycline (Dox), an analog of tetracycline (Fig. 5A, S3A–B Fig.). Upon Dox treatment, we examined the localization of ATF7-wt and ATF7-TA in specific cell-cycle phases and found that ATF7-wt and ATF7-TA were similarly localized to the nucleus in interphase, the cytoplasm from prophase to anaphase, and on mitotic chromosomes in telophase (Fig. 5B). These results suggest that the state of mitotic phosphorylation of ATF7 at Thr-51/Thr-53 does not affect the localization of ATF7.

Next, to examine whether the mitotic phosphorylation of ATF7 affected cell-cycle progression, we synchronized parental cells, ATF7-wt- and ATF7-TA-inducible cells with DTB, and treated them with Dox after release from DTB (Fig. 5C, 0 h). In the case of parental cells, 19% of cells progressed into M phase at 10.5 h as assessed by induction of the phosphorylation of histone H3 at Ser-10. Subsequently, 24% and 62% of cells divided and proceeded to enter G1 phase at 11.5 h and 12.5 h, respectively. Moreover, upon ATF7-wt induction, the rate of cell-cycle progression was similar to that of parental cells. However, upon ATF7-TA induction, only 2∼11% of cells progressed into M phase at 10.5 h. Subsequently, only 9∼14% and 16∼37% of cells divided and proceeded to enter G1 phase at 11.5 h and 12.5 h, respectively (Fig. 5C, S3C–D and S4 Figs.). To investigate in greater detail the effect of the phosphorylation state of ATF7 on the G2/M transition, we synchronized cells in late G2 phase using RO-3306, and released cells into M phase. For induction of ATF7-wt and ATF7-TA, cells were treated with Dox at 5 h before release from G2 arrest. We found that the expression of ATF7-TA, but not of ATF7-wt, inhibited progression into M phase even in the presence of endogenous ATF7 (Fig. 5D, S3E Fig.), which suggests that ATF7-TA acts as a dominant-negative protein. These results indicate that the mitotic phosphorylation of ATF7 is required for normal G2/M progression.

### Effect of ATF7 phosphorylation on Aurora signaling

**Figure 6:**
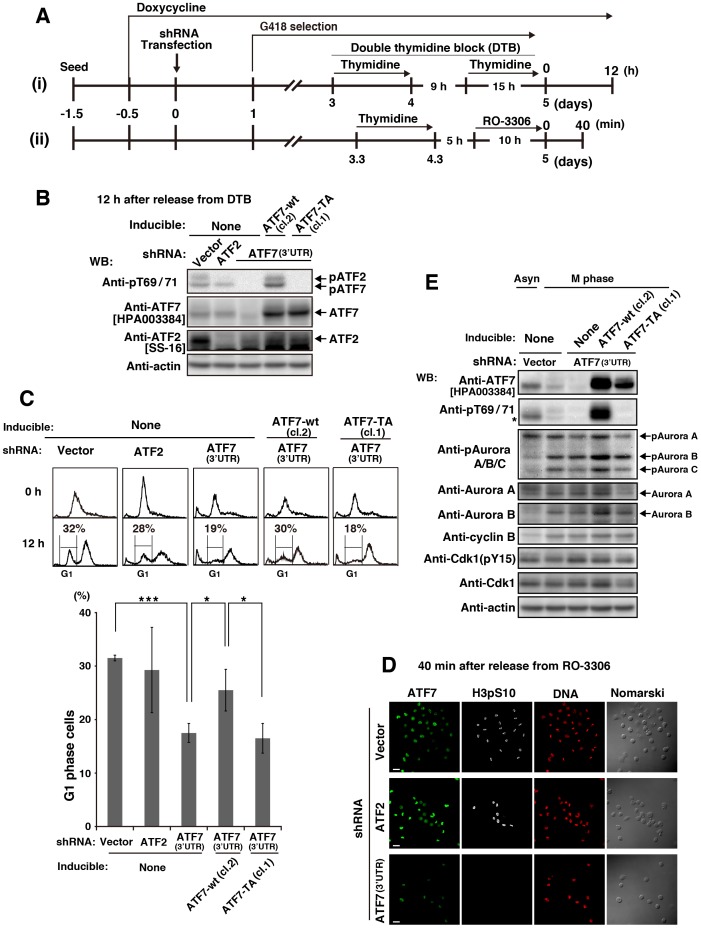
Involvement of ATF7 phosphorylation in Aurora signaling. (**A**) Schematic depiction of our knockdown-rescue experiments. Parental HeLa S3/TR, HeLa S3/TR/ATF7-wt (cl. 2), or HeLa S3/TR/ATF7-TA (cl.1) cells were treated with 1 µg/ml Dox for 12 h and then transfected with shRNAs. Knockdown cells selected using 600 µg/ml G418 in the presence of 1 µg/ml Dox were synchronized by (i) DTB or (ii) thymidine→RO-3306. (**B–D**) Knockdown cells were synchronized as described in (a)-(i) and collected 12 h after release from DTB. (**B**) Whole cell lysates were analyzed by WB. Full-length blots are presented in S12B Fig. (**C**) Cells were stained with PI for analyzing cell-cycle progression by flow cytometry (panels) and for quantitating G1-phase cells (graph). Values are means ± SD, n  = 4 independent experiments. Asterisks indicate the significant differences (*P<0.05; ***P<0.001), as calculated by Student’s *t*-test. (**D, E**) Knockdown cells were synchronized as described in (A)-(ii) and collected 40 min after release from RO-3306. (**D**) Cells were triply stained with anti-ATF7[HPA003384] and anti-histone H3pS10 antibodies (for M phase) and PI (for DNA). Cells stained with anti-histone H3pS10 antibody are pseudo-colored as white. Scale bars, 40 µm. (**E**) Whole cell lysates were analyzed by WB. *, a nonspecific band. Full-length blots are presented in S13 Fig.

Although G2/M progression was inhibited by ATF7-TA expression (Fig. 5C–D, S3C–E and S4 Figs.), it was unclear how phosphorylation of ATF7 was involved in G2/M progression. To investigate the effect of ATF7 phosphorylation on M phase, we wished to perform rescue experiments using ATF7-knockdown cells. However, considering that ATF7 knockdown inhibited cell proliferation (Fig. 1C, E, S1F Fig.), we assumed that ATF7-knockdown cells are not capable of undergoing cell-cycle synchronization because of poor cell-cycle progression. Accordingly, the expression of ATF7-wt and ATF7-TA was induced by Dox addition before endogenous ATF7 proteins were knocked down, and the cells were subsequently synchronized in the presence of Dox using the DTB method (Fig. 6A–C, S5C–E Fig.) or the thymidine→RO-3306 method (Fig. 6A, D, and E, S5B, F, and G Fig.). We confirmed the successful knockdown of endogenous ATF7 and ATF2 (Fig. 6B, E, S5A, B, F, And G Fig., see also Fig. 1B). Because transfection of shATF7(3′UTR) appeared to act mainly in M phase (Fig. 1E), inducible expression of ATF7-wt and ATF7-TA, none of which contain the 3′-untranslated region, was carried out under conditions in which shATF7(3′UTR) was transfected (Fig. 6B, S5A–B Fig.). To examine whether ATF7 knockdown affected the number of daughter cells after mitosis, we measured the percentage of G1-phase cells using a flow cytometer 12 h after release from DTB (Fig. 6C, S5E Fig., see Fig. 1E). We found that ATF7 knockdown decreased the number of G1 cells and that the inducible expression of ATF7-wt, but not ATF7-TA, in ATF7-knockdown cells recovered the number of G1 cells (Fig. 6C, S5E Fig.). Furthermore, ATF7 knockdown decreased the number of viable cells, accompanied by an increase in the number of subG1 cells, and this ATF7-knockdown-induced apoptosis was alleviated by the inducible expression of ATF7-wt, but not ATF7-TA (S5C–D Fig.). These results suggest that phosphorylation of ATF7 is important for cell-cycle progression and cell viability.

To examine whether ATF7-knockdown cells were capable of entering M phase, these cells were synchronized in G2/M phase before completion of ATF7 knockdown using the method depicted in Fig. 6A-(**ii**) and co-immunostained for both endogenous ATF7 and Ser10-phosphorylated histone H3 as an M phase marker was carried out. It has to be emphasized that phosphorylation of histone H3 at Ser-10 was not detectable in ATF7-knockdown cells (Fig. 6D). Because Aurora kinase phosphorylates histone H3 at Ser-10 [42]–[45], we examined whether the phosphorylation of ATF7 played a role in Aurora signaling. Western blot analysis showed that the phosphorylation and protein levels of Aurora A, B, and C, in particular Aurora A, were reduced in G2/M phase after ATF7 knockdown (Fig. 6E, S5F–G Fig.). Importantly, the inducible expression of ATF7-wt, but not ATF7-TA, in ATF7-knockdown cells restored the phosphorylation and protein levels of Aurora kinases to normal M-phase levels. These results indicate that ATF7 phosphorylation plays a role in Aurora signaling, which is known to be essential for G2/M transition.

## Discussion

**Figure 7:**
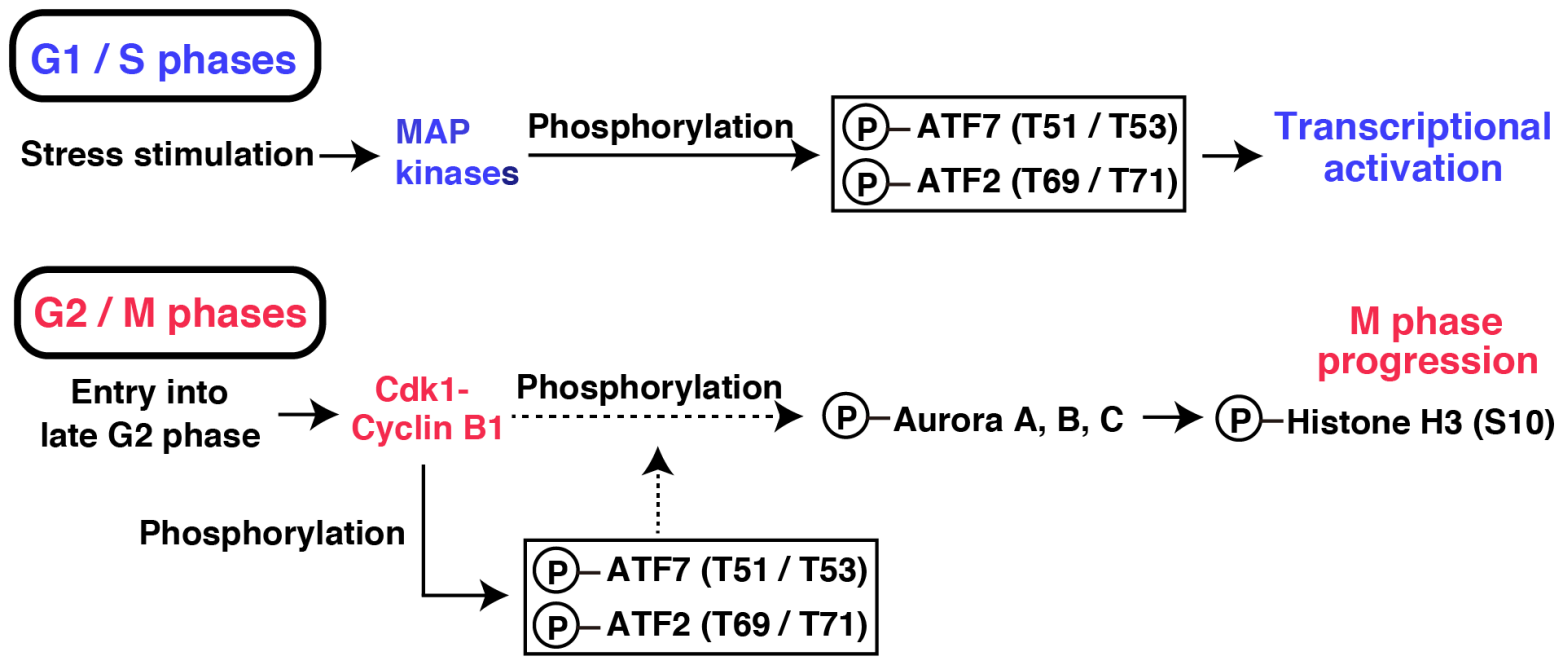
Differential roles for the phosphorylation of ATF7 and ATF2 in G1/S phases and G2/M phases. In G1 and S phases, ATF7 and ATF2 are phosphorylated at Thr-51 and Thr-53 and at Thr-69 and Thr-71, respectively, by stress-activated MAP kinases, to induce their transcriptional functions. During late G2 and M phases, the Thr-51/Thr-53 on ATF7 and the Thr-69/Thr-71 on ATF2 are phosphorylated by Cdk1-cyclin B1, which promotes M-phase entry via the stabilization of Aurora kinases.

In the present study, we have identified for the first time ATF2 and ATF7 as novel M phase substrates of the Cdk1-cyclin B1 complex. Mitotic phosphorylation of human ATF2 takes place on Thr-69/Thr-71 and on Thr-51/Thr-53 for human ATF7 from early prophase to anaphase. In agreement with a role for ATF2 and ATF7 in enabling passage through M phase, knockdown of both endogenous ATF2 and ATF7 inhibited cell proliferation and cell-cycle progression through M phase, but the effect of ATF7 knockdown was more severe compared with that of ATF2. We have also shown that the inducible expression of mitotically nonphosphorylatable ATF7-TA, but not ATF7-wt, inhibited G2/M progression, despite the presence of endogenous ATF7. Knockdown rescue experiments revealed that the mitotic phosphorylation of human ATF7 by the Cdk1-cyclin B1 complex plays an important role in enabling Aurora signaling and mitotic entry. Our results also suggest that the threonine-phosphorylation of ATF7 and ATF2 in G1/S phases versus G2/M phases play differential functional roles (Fig. 7).

A growing body of evidence demonstrates that phosphorylation of ATF2 at Thr-69/Thr-71 and of ATF7 at Thr-51/Thr-53 in the N-terminal activation domains is required for their transcriptional activation upon exposure of G1/S-phase cells to stress stimuli [9]–[13], [16], [17]. However, the role of ATF2 and ATF7 in G2/M phases has not yet been reported. Intriguingly, the protein structure of human ATF2 is highly homologous to that of human ATF7 in the N-terminal activation domain, which contains the threonine-phosphorylation sites, and in the DNA-binding domain (Fig. 3A, S6A Fig.). ATF2 is maintained in an inactive form by an intramolecular interaction between the N-terminal activation domain and the DNA-binding domain, and activators of ATF2 disrupt this interaction to activate transcription [46]. The activators of ATF2 include (i) regulatory proteins, such as adenovirus E1A, HTLV-I Tax, HMG1, and c-Jun, which are able to interact with the DNA-binding domain; and (ii) threonine phosphorylation by stress-activated MAP kinases, which increases the activity of the DNA-binding domain, both of which may be coordinately regulated [17], [46]–[52]. It is well known that activation of the Cdk1-cyclin B1 complex is required for G2/M entry and progression, and that the Cdk1-cyclin B1 complex is activated from late G2 phase to late anaphase and phosphorylates various mitotic substrates [53], [54]. Considering that the two threonine residues on human ATF2 and human ATF7 that are phosphorylated by the Cdk1-cyclin B1 complex are identical to the respective sites that are phosphorylated by MAP kinases (Fig. 2, 3), their mitotic phosphorylation may play a role in regulating dynamic events during M phase. Furthermore, we hypothesized that ATF2 and ATF7 directly or indirectly interact with nucleocytoplasmic or cytoplasmic proteins, rather than chromosomes, during M phase, because mitotically phosphorylated ATF2 and ATF7 do not appear to localize to chromosomal DNA, but are dispersed throughout the nucleocytoplasm/cytoplasm from early prophase to anaphase (Fig. 4D–E). Thus, these novel functions of ATF2 and ATF7 may be found in the nucleocytoplasm/cytoplasm during G2/M progression, unlike those of AP-1-dependent transcription factor during G1 and S phases [9]–[12].

Similar to human ATF2, human ATF7 was phosphorylated by the Cdk1-cyclin B1 complex during M phase (Fig. 2, 3, and 4A). However, knockdown of human ATF7 inhibited cell proliferation and cell-cycle progression much more strongly than did that of human ATF2 (Fig. 1C–E, S1F Fig.). Because human ATF7 exhibits relatively low homology to human ATF2 in the middle and the C-terminal regions (S6A Fig.), it is reasonable to assume that the role of human ATF7 in M phase is somewhat different from that of human ATF2. Moreover, given that double knockdown of ATF2 and ATF7 yielded strong inhibition of mitotic progression compared with single knockdown of ATF2 or ATF7 (S1F Fig.), ATF2 and ATF7 might play a synergistic role in mitotic progression. Notably, the human ATF2 and human ATF7 genes generate 13 and six transcript variants, respectively. Our knockdown construct against human ATF2 (shATF2) targeted the common sequence among all of the 13 transcript variants of human ATF2 [28], [29] (see Materials and Methods). Regarding ATF7 knockdown, one of our knockdown constructs against human ATF7 [shATF7(3′UTR)] targeted the human ATF7 transcript variant 2, transcript variant 3, and an uncharacterized long noncoding RNA, and the other knockdown construct [shATF7(CDS)] targeted human ATF7 transcript variant 2, transcript variant 3, and long noncoding RNA transcript variant 6 (S1A Fig., see Materials and Methods). Despite the induction of partial arrest in G1 phase by shATF7(CDS) (Fig. 1E), both shATF7(CDS) and shATF7(3′UTR) are fully capable of knocking down human ATF7 proteins and of inhibiting cell-cycle progression from M phase to G1 phase (Fig. 1B, S1 Fig.). Considering that the inducible expression of ATF7-TA inhibited G2/M progression, most likely because of a dominant-negative effect (Fig. 5C–D S3 and S4 Figs.), it should be emphasized that the mitotic phosphorylation of human ATF7 proteins, rather than human ATF2 proteins, is important for cell-cycle progression from M phase to G1 phase (Fig. 1E).

Mice bearing a deletion of the DNA-binding domain of the ATF7 gene are viable, and no severe abnormalities are observed in adult animals [2], [41]. Regarding ATF2 knockout mice, the deletion of the DNA-binding domain of the ATF2 gene invariably leads to death at birth [8]. Knock-in mutant mice in which the two threonine-phosphorylation sites located in the N-terminal ATF2 activation domain are mutated into alanine also die shortly after birth [2]. In contrast with the single deletions, ATF2/ATF7 double deletion results in severe hypoplasia of the embryonic heart and liver, which leads to death between embryonic days 11.5 and 12.5 [2]. This demonstrates that mouse ATF2 and mouse ATF7 share essential functions during embryonic development [2], [17]. Nonetheless, primary fetal liver cells prepared from ATF2/ATF7 double knockout mice are able to grow over 7 days in culture, although the cells exhibit reduced rates of cell proliferation after 2∼3 days of culture, as well as an increase in the number of apoptotic cells [2]. Collectively, we assumed that mouse ATF7 does not have a significant effect on cell proliferation compared with human ATF7. Human ATF7 may play a more complex role in cell signaling than does mouse ATF7 because human ATF7 has six transcript variants and the structure of the human ATF7 protein contains an additional C-terminal sequence compared with that of mouse ATF7 (S1A and S6B Fig.). In fact, we have not yet succeeded in establishing stable ATF7 knockdown HeLa S3 cell lines, which may be attributable to the fact that knockdown of human ATF7 strongly inhibits cell proliferation (Fig. 1C). To overcome this problem, we introduced an EBNA1-based episomal vector encoding an shRNA and selected knockdown cells using G418 in a short span of time (Fig. 1A, E, and 6A). Our knockdown method has the advantage of avoiding the preparation of a single-cell clone on the grounds that a single-cell clone is, to some extent, idiosyncratic and may have undergone compensatory adaptations or mutations.

ATF2 and ATF7 can form homodimers and heterodimers with other AP-1 transcription factors [17], [18]. In fact, we showed that ATF2 is co-immunoprecipitated with ATF7 from Triton X-100 lysates of asynchronous and M-phase cells (Fig. 4A), which is supported by *in* *vitro* data [55]. N-terminal phosphorylation of ATF2 at Thr-69/Thr-71 by MAP kinases protects ATF2 from ubiquitination and degradation [56], [57]. Furthermore, ATF7 knockdown affects the level of the ATF2 protein, and vice versa (Fig. 1B, 6B, S1B-E Fig.). Taken together, these results suggest that ATF2 and ATF7 actually interact with each other *in* *vivo* to regulate their stabilization.

Intriguingly, we showed that mitotic phosphorylation of ATF7 is involved in Aurora signaling (Fig. 6E, S5F–G Fig.). The Aurora family consists of three members, Aurora A, B, and C. Aurora A is an oncogene in a variety of cancers and plays a role in centrosome maturation for G2/M transition [58]. The disruption of Aurora A function delays mitotic entry [59], [60]. Since inhibition of Aurora kinases is also known to induce apoptosis [61], down-regulation of Aurora kinase signaling by ATF7 knockdown (Fig. 6) may explain an increase in the presence of cells in subG1 phase (Fig. 1D) following G2/M-phase arrest of cells in which ATF7 was knocked down (Fig. 1C, S1F Fig.). Inhibition of the kinase activity of Cdk1 induces Aurora A inactivation, even though Cdk1 does not directly phosphorylate Aurora A [62]. Activated Aurora B phosphorylates histone H3 at Ser-10 [42]–[45]. However, once Cdk1 is activated, mitotic phosphorylation of ATF7 precedes phosphorylation of histone H3 at Ser-10 (Fig. 4E, F), which suggests that ATF7 is located upstream of Aurora kinases. Thus, we assumed that the mitotic phosphorylation of ATF7 by the Cdk1-cyclin B1 complex promotes the activation of Aurora kinases for mitotic entry via the stabilization of Aurora kinases. In addition, to examine the effect of proteasome inhibition on ATF7-mediated M-phase progression, we treated parental cells, ATF7-wt- or ATF7-TA-inducible cells with the proteasome inhibitor MG132 at the onset of mitosis. Treatment with MG132 almost fully blocked entry of ATF7-TA-induced cells into M phase (S5H Fig.), which suggests that ATF7-TA-mediated inhibition of M-phase progression is augmented by MG132 treatment. Considering that MG132 blocks entry into and exit from mitosis through intricate mechanisms [63], [64], we hypothesized that the mitotic phosphorylation of ATF7 promotes Aurora signaling at the onset of M phase in a manner dependent on proteasome activity. Although we have not yet detected any physical associations between ATF7 and the phosphorylated Aurora kinases, INCENP or TPX2, it is now of interest to determine precisely how the mitotic phosphorylation of ATF7 is involved in Aurora signaling.

## Supporting Information

S1 Fig **Inhibition of cell-cycle progression by shATF7(3**′**UTR) and shATF7(CDS).** **(A)** Schematic representation of the exon-intron structure of the human ATF7 transcript variants. At least six transcript variants, four isoforms, and two noncoding RNAs, are found for the human ATF7 gene (NM_006856.2, NM_001130060.1, NM_001206682.1, NM_001206683.1, NR_073163.1, NR_046221.1). The red arrows run through the target sites of shRNA. Numbers, the number of base pairs (bp); boxes, exon (Ex); *, start codon; x, stop codon; pA, poly A tail; CDS, coding sequence; 3′UTR, 3′-untranslated region. **(B, C)** Cells were transfected with control vector, ATF2 shRNA, or ATF7 shRNA(3′UTR) and collected on day 5 (B) or days 3.5–6 (C). **(D, E)** Cells were transfected with control vector, ATF2 shRNA, ATF7 shRNA(CDS), or double-knockdown shRNAs[ATF2 and ATF7(CDS)] and collected on day 7 (D) or day 9 (E). Whole cell lysates were analyzed by WB. Full-length blots are presented in S14 and S15 Figs. **(F)** Cells transfected with control vector, ATF2 shRNA, ATF7 shRNA(3′UTR), ATF7 shRNA(CDS), or 6 double knockdown shRNAs[ATF2 and ATF7(CDS)] were synchronized using double thymidine block (DTB) in the presence of 600 µg/ml G418, and released into thymidine-free medium for 12 h. M-phase cells were counted.
(TIF)

S2 Fig **Effect of ATF2 or ATF7 knockdown on the percentages of G1-phase cells. (A, B)** Knockdown cells were synchronized as described in Fig. 1E. **(A)** Cells were collected 10∼12 h after release from DTB and stained with PI for analyzing cell cycle progression by flow cytometry (left panels). The percentages of G1-phase cells were compared between 10 h and 12 h after release from DTB (right graph). **(B)** Cells were collected 12 h after release from DTB and stained with PI for analyzing cell cycle progression by flow cytometry (left panels). The percentages of G1-phase cells were quantitated. Values are means ± SD, n = 3 independent experiments (right graph).
(TIF)

S3 Fig **Effect of inducible ATF7-TA expression on M-phase entry.** **(A, B)** Parental HeLa S3/TR, HeLa S3/TR/ATF7-wt (cl.2), or HeLa S3/TR/ATF7-TA (cl.1) cells were cultured with or without 1 µg/ml Dox for the indicated times. Whole cell lysates were analyzed by WB. Full-length blots are presented in S16 Fig. **(C)** Cells were synchronized using DTB and released into thymidine-free medium for 11 h in the presence of 1 µg/ml Dox. M-phase cells were counted. **(D, E)** Cells were stained with anti-histone H3pS10 antibody (for M phase) and PI for analyzing cell-cycle progression by flow cytometry. **(D)** Parental HeLa S3/TR, HeLa S3/TR/ATF7-wt (cl.2), or HeLa S3/TR/ATF7-TA (cl.1, cl.2, cl.3) cells were synchronized using DTB and released into thymidine-free medium containing 1 µg/ml Dox for 10–12 h. Exp.1–5 were five independent experiments. **(E)** HeLa S3/TR/ATF7-wt (cl.2) or HeLa S3/TR/ATF7-TA (cl.1) cells were cultured in the presence of 9 µM RO-3306 for 10 h and treated with 1 µg/ml Dox for the last 5 h. The cells arrested at G2 phase were released into RO-3306-free medium containing 1 µg/ml Dox and incubated for 0, 10, and 20 min.
(TIF)

S4 Fig **Histograms of different clones for** **Fig. 5C** **.** Parental HeLa S3/TR, HeLa S3/TR/ATF7-wt (three independent inducible clones: cl.1, cl.2, and cl.3), or HeLa S3/TR/ATF7-TA (three independent inducible clones: cl.1, cl.2, and cl.3) cells were synchronized using DTB and released into thymidine-free medium containing 1 µg/ml Dox for 10.5∼12.5 h. Two-dimensional histograms (DNA vs histone H3pS10) are presented together with DNA histograms, and the percentages of cells in G1 and M phases were measured.
(TIF)

S5 Fig **Knockdown/rescue of mitotic phosphorylation of ATF7: cell cycle progression and apoptosis. (A)** Parental HeLa S3/TR, HeLa S3/TR/ATF7-wt (cl.2), or HeLa S3/TR/ATF7-TA (cl.1) cells were transfected with control vector, ATF2 shRNA, or ATF7 shRNA(3′UTR) and collected on day 5. **(B)** Parental HeLa S3/TR and HeLa S3/TR/ATF7-wt (cl.2) cells transfected with vector control or shATF7 were arrested at G2 phase with or without 1 µg/ml Dox. Subsequently, knockdown cells were released into RO-3306-free medium for 40 min with or without 1 µg/ml Dox. **(C, D)** Knockdown cells were synchronized as described in Fig. 6A-(i), and viable cells were counted 12 h after release from DTB (C). Cells were stained with PI for analyzing cell cycle progression by flow cytometry and for quantitating subG1-phase cells (D). Values are means ± SD, n = 3 independent experiments. Asterisks indicate the significant differences (*P<0.05; **P<0.01; ***P<0.001; NS, not significant), as calculated by Student’s t-test. **(E)** Cells were analyzed by flow cytometry as described in Fig. 6C. Exp.1, Exp.2, and Exp.3 were three independent experiments. **(F, G)** Cells were synchronized as described in Fig. 6A-(ii). Whole cell lysates were analyzed by WB. Full-length blots are presented in S17 and S18 Figs. (F) and (G) were independent experiments. **(H)** Inducible overexpression of ATF7-wt and ATF7-TA was performed without ATF7 knockdown, as described in Fig. 5C. In brief, parental HeLa S3/TR, HeLa S3/TR/ATF7-wt (cl.2), or HeLa S3/TR/ATF7-TA (cl.1) cells were synchronized using DTB and released into thymidine-free medium containing 1 µg/ml Dox for 10 or 11 h. At 10 h after DTB release, cells were treated for an additional 1 h in the presence or absence of 10 µM MG132, together with 1 µg/ml Dox. Cells were stained with anti-histone H3pS10 antibody (for M phase) and PI for analyzing cell cycle progression by flow cytometry.
(TIF)

S6 Fig **Amino acid sequence alignment of ATF2 and ATF7. (A)** The longest isoform of human ATF2 (isoform 1: NP_001243019.1) and the longest isoform of human ATF7 (isoform 2: NM_006856.2) are compared, and amino acid sequence identity (%) is shown. **(B)** Amino acid sequence alignment of mouse and human ATF7. The mouse ATF7 protein and the longest isoform of human ATF7 (isoform 2: NM_006856.2) are compared, because the mouse ATF7 gene generates a single transcript (NM_146065.1).
(TIF)

S7 Fig **Full-length blots for** **Fig. 1B** **and** **Fig. 2A** **.**
(TIF)

S8 Fig **Full-length blots for** **Fig. 2C** **.**
(TIF)

S9 Fig **Full-length blots for** **Fig. 2D** **.**
(TIF)

S10 Fig **Full-length blots for** **Fig. 3B, 3C, 3D, and 3E** **.**
(TIF)

S11 Fig **Full-length blots for** **Fig. 4A, 4F, and 4G** **.**
(TIF)

S12 Fig **Full-length blots for** **Fig. 5A** **and** **6B** **.**
(TIF)

S13 Fig **Full-length blots for** **Fig. 6E** **.**
(TIF)

S14 Fig **Full-length blots for S1B, C Fig.**
(TIF)

S15 Fig **Full-length blots for S1D, E Fig.**
(TIF)

S16 Fig **Full-length blots in S3A, B Fig.**
(TIF)

S17 Fig **Full-length blots for S5A, B, and F Fig.**
(TIF)

S18 Fig **Full-length blots for S5G Fig.**
(TIF)
