# Supplementary figures and images for: Cdk1-Mediated Phosphorylation of Human ATF7 at Thr-51 and Thr-53 Promotes Cell-Cycle Progression into M Phase
Source: PLoS One. 2014 Dec 29;9(12):e116048. doi: 10.1371/journal.pone.0116048 (PMC4278844; doi:10.1371/journal.pone.0116048)

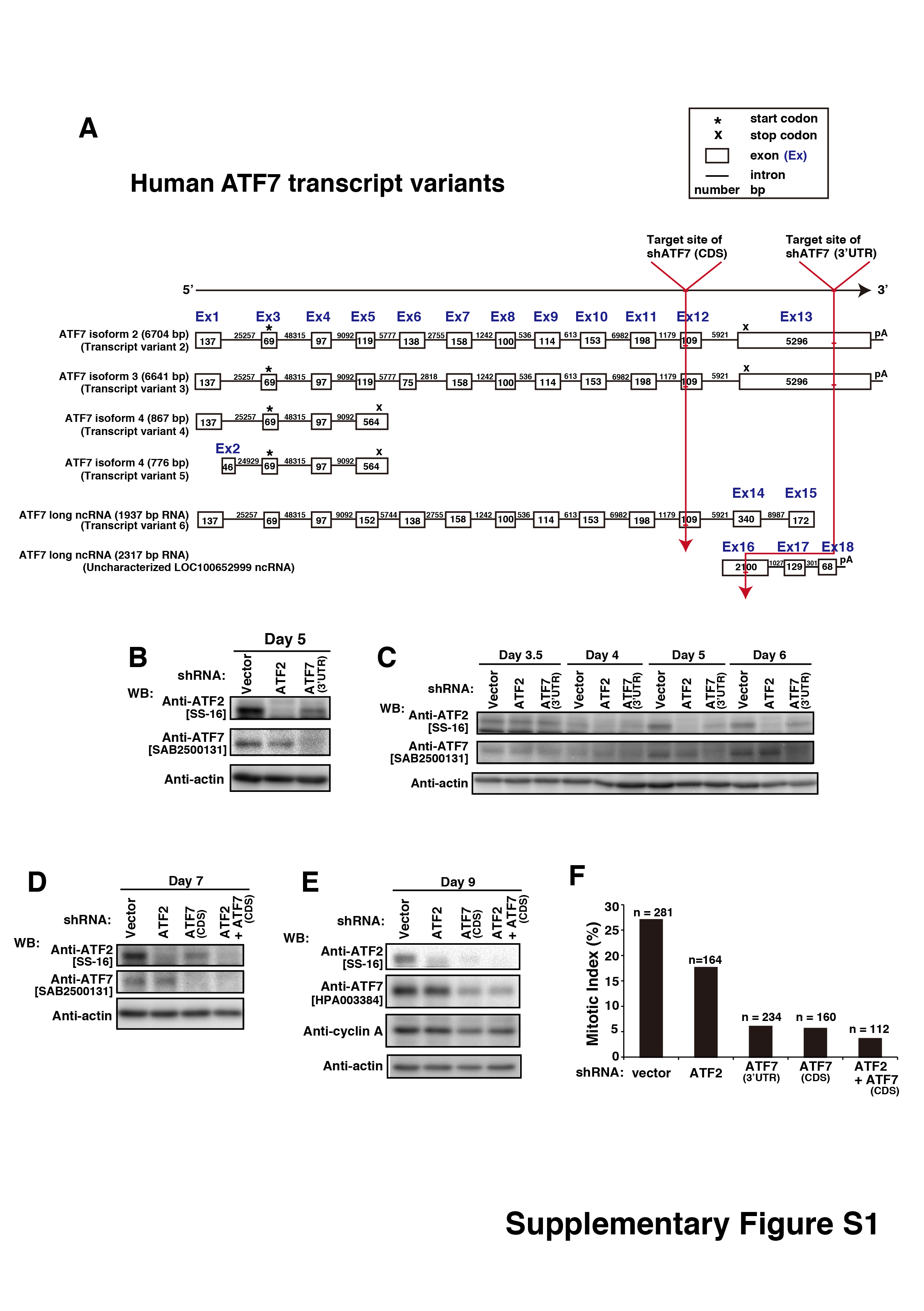

Supplement: S1 Fig — Inhibition of cell-cycle progression by shATF7(3′UTR) and shATF7(CDS). (A) Schematic representation of the exon-intron structure of the human ATF7 transcript variants. At least six transcript variants, four isoforms, and two noncoding RNAs, are found for the human ATF7 gene (NM_006856.2, NM_001130060.1, NM_001206682.1, NM_001206683.1, NR_073163.1, NR_046221.1). The red arrows run through the target sites of shRNA. Numbers, the number of base pairs (bp); boxes, exon (Ex); *, start codon; x, stop codon; pA, poly A tail; CDS, coding sequence; 3′UTR, 3′-untranslated region. (B, C) Cells were transfected with control vector, ATF2 shRNA, or ATF7 shRNA(3′UTR) and collected on day 5 (B) or days 3.5–6 (C). (D, E) Cells were transfected with control vector, ATF2 shRNA, ATF7 shRNA(CDS), or double-knockdown shRNAs[ATF2 and ATF7(CDS)] and collected on day 7 (D) or day 9 (E). Whole cell lysates were analyzed by WB. Full-length blots are presented in S14 and S15 Figs. (F) Cells transfected with control vector, ATF2 shRNA, ATF7 shRNA(3′UTR), ATF7 shRNA(CDS), or 6 double knockdown shRNAs[ATF2 and ATF7(CDS)] were synchronized using double thymidine block (DTB) in the presence of 600 µg/ml G418, and released into thymidine-free medium for 12 h. M-phase cells were counted. (TIF) [file pone.0116048.s001.tif]

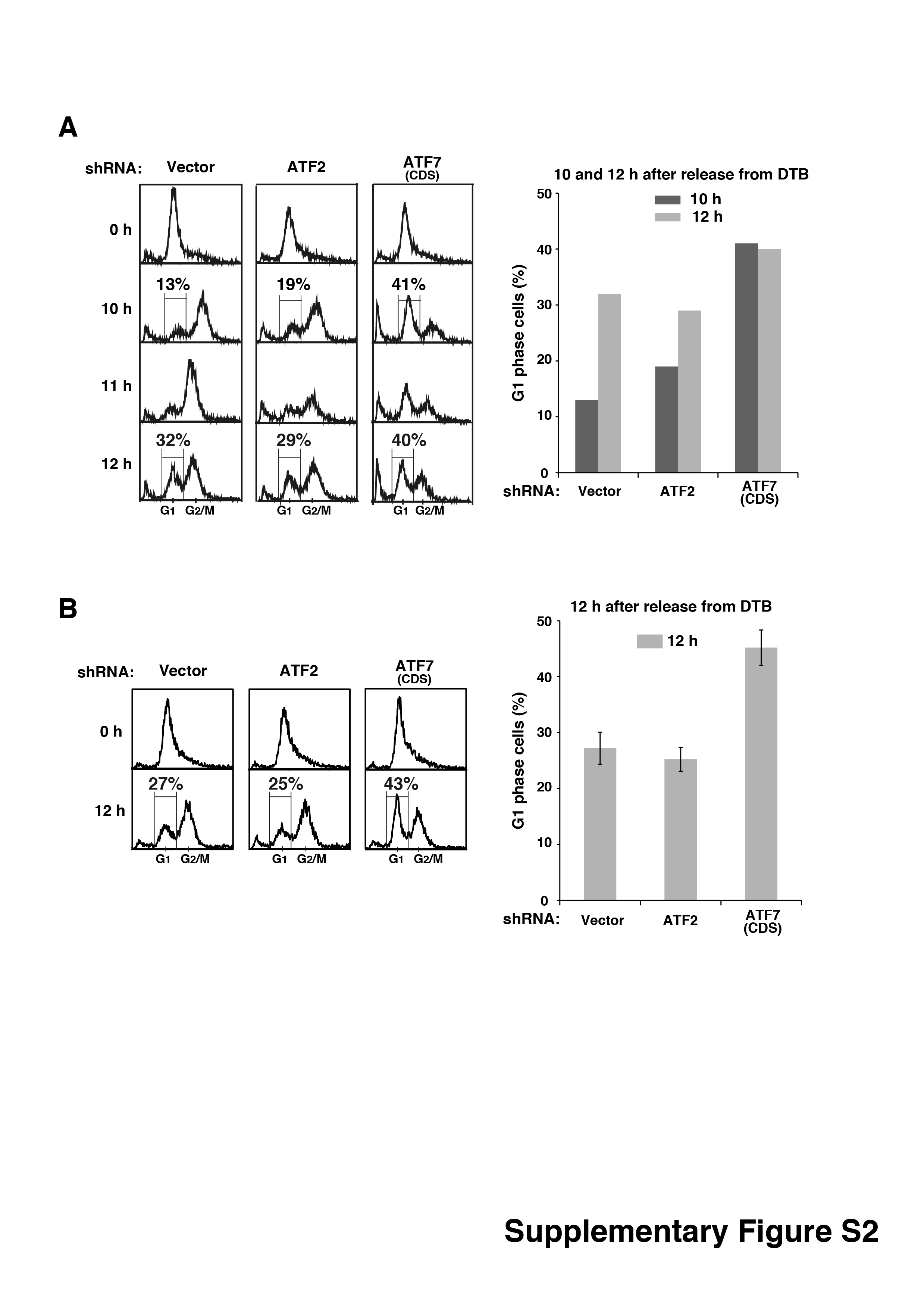

Supplement: S2 Fig — Effect of ATF2 or ATF7 knockdown on the percentages of G1-phase cells. (A, B) Knockdown cells were synchronized as described in Fig. 1E. (A) Cells were collected 10∼12 h after release from DTB and stained with PI for analyzing cell cycle progression by flow cytometry (left panels). The percentages of G1-phase cells were compared between 10 h and 12 h after release from DTB (right graph). (B) Cells were collected 12 h after release from DTB and stained with PI for analyzing cell cycle progression by flow cytometry (left panels). The percentages of G1-phase cells were quantitated. Values are means ± SD, n = 3 independent experiments (right graph). (TIF) [file pone.0116048.s002.tif]

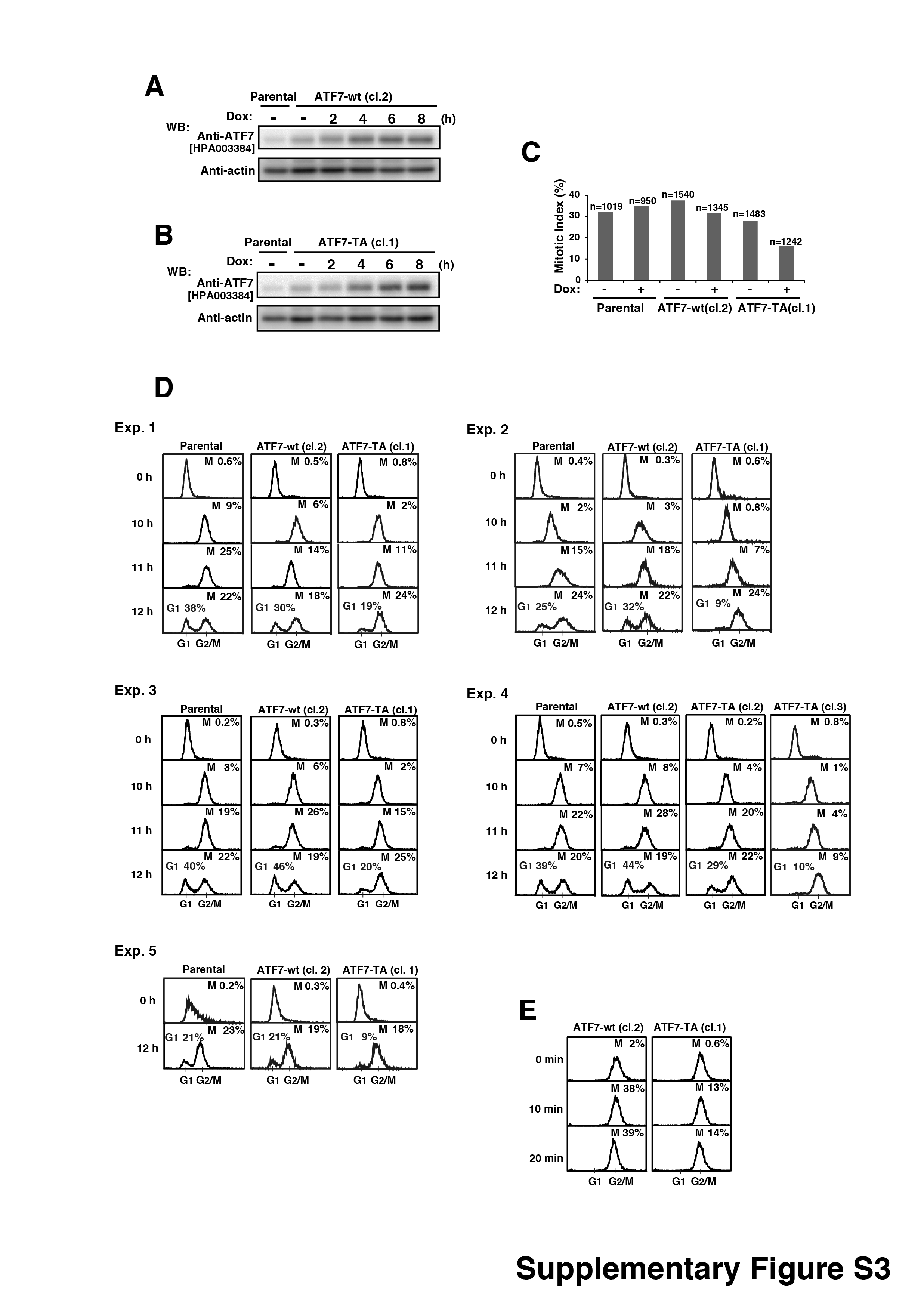

Supplement: S3 Fig — Effect of inducible ATF7-TA expression on M-phase entry. (A, B) Parental HeLa S3/TR, HeLa S3/TR/ATF7-wt (cl.2), or HeLa S3/TR/ATF7-TA (cl.1) cells were cultured with or without 1 µg/ml Dox for the indicated times. Whole cell lysates were analyzed by WB. Full-length blots are presented in S16 Fig. (C) Cells were synchronized using DTB and released into thymidine-free medium for 11 h in the presence of 1 µg/ml Dox. M-phase cells were counted. (D, E) Cells were stained with anti-histone H3pS10 antibody (for M phase) and PI for analyzing cell-cycle progression by flow cytometry. (D) Parental HeLa S3/TR, HeLa S3/TR/ATF7-wt (cl.2), or HeLa S3/TR/ATF7-TA (cl.1, cl.2, cl.3) cells were synchronized using DTB and released into thymidine-free medium containing 1 µg/ml Dox for 10–12 h. Exp.1–5 were five independent experiments. (E) HeLa S3/TR/ATF7-wt (cl.2) or HeLa S3/TR/ATF7-TA (cl.1) cells were cultured in the presence of 9 µM RO-3306 for 10 h and treated with 1 µg/ml Dox for the last 5 h. The cells arrested at G2 phase were released into RO-3306-free medium containing 1 µg/ml Dox and incubated for 0, 10, and 20 min. (TIF) [file pone.0116048.s003.tif]

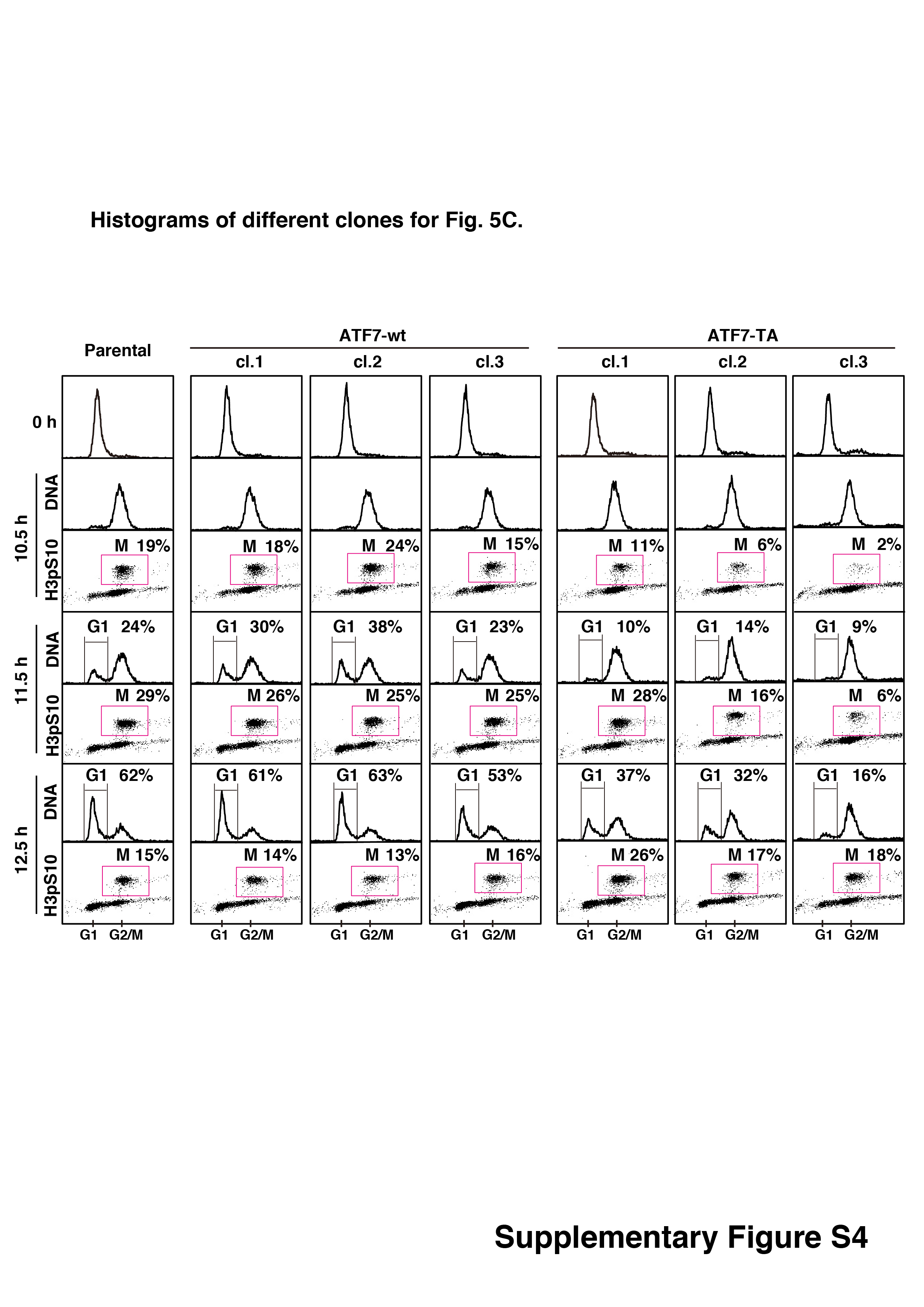

Supplement: S4 Fig — Histograms of different clones for Fig. 5C . Parental HeLa S3/TR, HeLa S3/TR/ATF7-wt (three independent inducible clones: cl.1, cl.2, and cl.3), or HeLa S3/TR/ATF7-TA (three independent inducible clones: cl.1, cl.2, and cl.3) cells were synchronized using DTB and released into thymidine-free medium containing 1 µg/ml Dox for 10.5∼12.5 h. Two-dimensional histograms (DNA vs histone H3pS10) are presented together with DNA histograms, and the percentages of cells in G1 and M phases were measured. (TIF) [file pone.0116048.s004.tif]

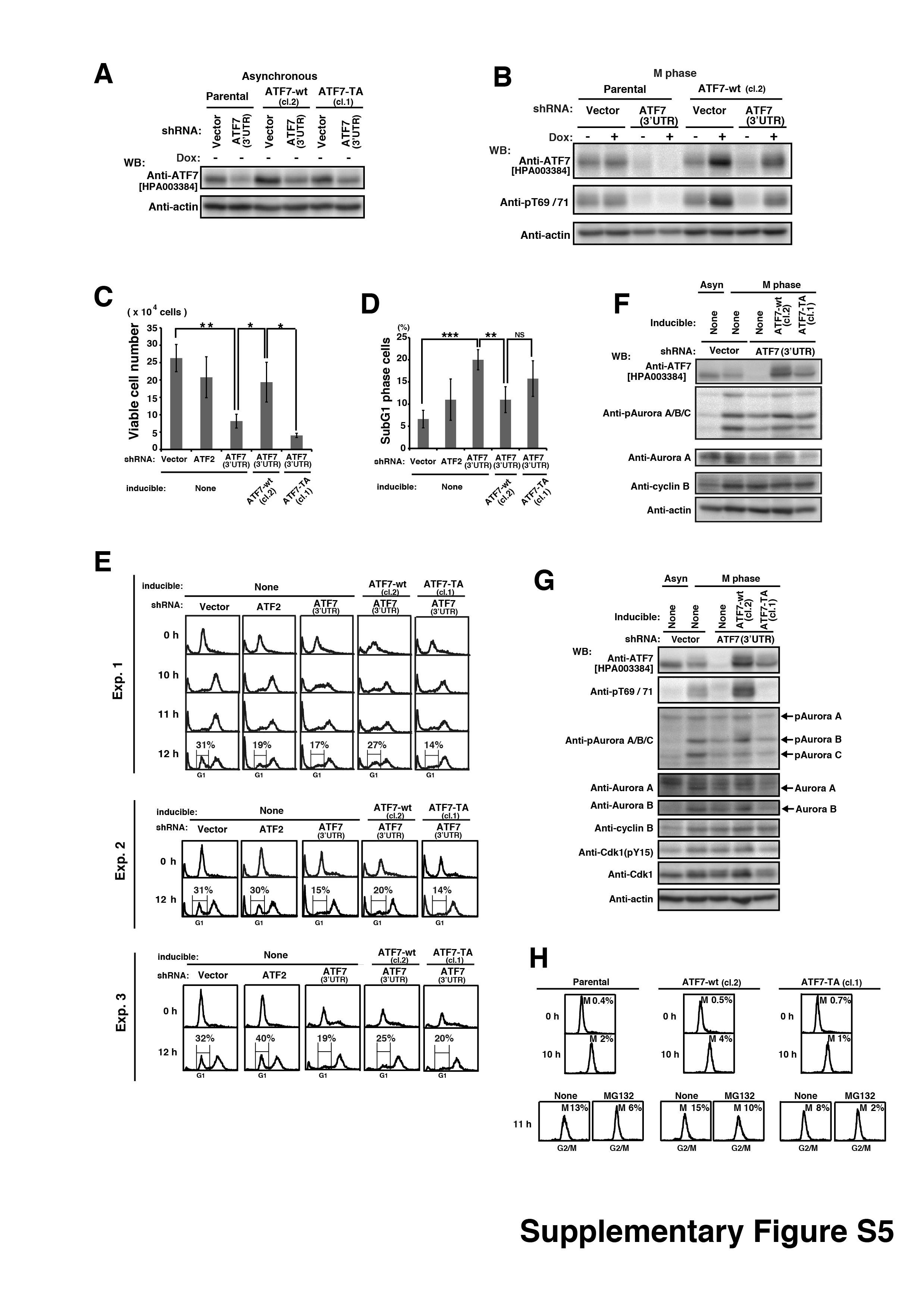

Supplement: S5 Fig — Knockdown/rescue of mitotic phosphorylation of ATF7: cell cycle progression and apoptosis. (A) Parental HeLa S3/TR, HeLa S3/TR/ATF7-wt (cl.2), or HeLa S3/TR/ATF7-TA (cl.1) cells were transfected with control vector, ATF2 shRNA, or ATF7 shRNA(3′UTR) and collected on day 5. (B) Parental HeLa S3/TR and HeLa S3/TR/ATF7-wt (cl.2) cells transfected with vector control or shATF7 were arrested at G2 phase with or without 1 µg/ml Dox. Subsequently, knockdown cells were released into RO-3306-free medium for 40 min with or without 1 µg/ml Dox. (C, D) Knockdown cells were synchronized as described in Fig. 6A-(i), and viable cells were counted 12 h after release from DTB (C). Cells were stained with PI for analyzing cell cycle progression by flow cytometry and for quantitating subG1-phase cells (D). Values are means ± SD, n = 3 independent experiments. Asterisks indicate the significant differences (*P<0.05; **P<0.01; ***P<0.001; NS, not significant), as calculated by Student’s t-test. (E) Cells were analyzed by flow cytometry as described in Fig. 6C. Exp.1, Exp.2, and Exp.3 were three independent experiments. (F, G) Cells were synchronized as described in Fig. 6A-(ii). Whole cell lysates were analyzed by WB. Full-length blots are presented in S17 and S18 Figs. (F) and (G) were independent experiments. (H) Inducible overexpression of ATF7-wt and ATF7-TA was performed without ATF7 knockdown, as described in Fig. 5C. In brief, parental HeLa S3/TR, HeLa S3/TR/ATF7-wt (cl.2), or HeLa S3/TR/ATF7-TA (cl.1) cells were synchronized using DTB and released into thymidine-free medium containing 1 µg/ml Dox for 10 or 11 h. At 10 h after DTB release, cells were treated for an additional 1 h in the presence or absence of 10 µM MG132, together with 1 µg/ml Dox. Cells were stained with anti-histone H3pS10 antibody (for M phase) and PI for analyzing cell cycle progression by flow cytometry. (TIF) [file pone.0116048.s005.tif]

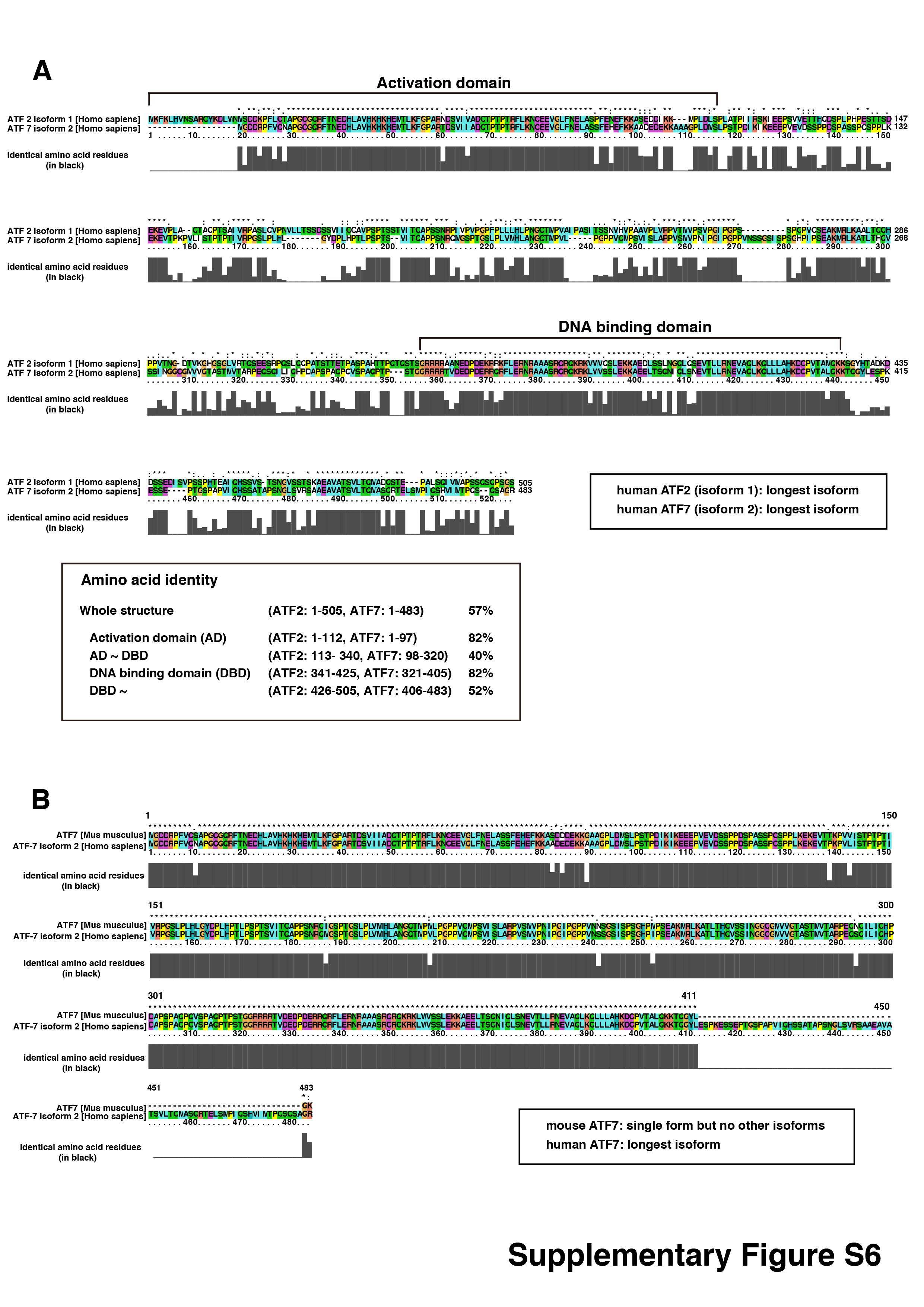

Supplement: S6 Fig — Amino acid sequence alignment of ATF2 and ATF7. (A) The longest isoform of human ATF2 (isoform 1: NP_001243019.1) and the longest isoform of human ATF7 (isoform 2: NM_006856.2) are compared, and amino acid sequence identity (%) is shown. (B) Amino acid sequence alignment of mouse and human ATF7. The mouse ATF7 protein and the longest isoform of human ATF7 (isoform 2: NM_006856.2) are compared, because the mouse ATF7 gene generates a single transcript (NM_146065.1). (TIF) [file pone.0116048.s006.tif]

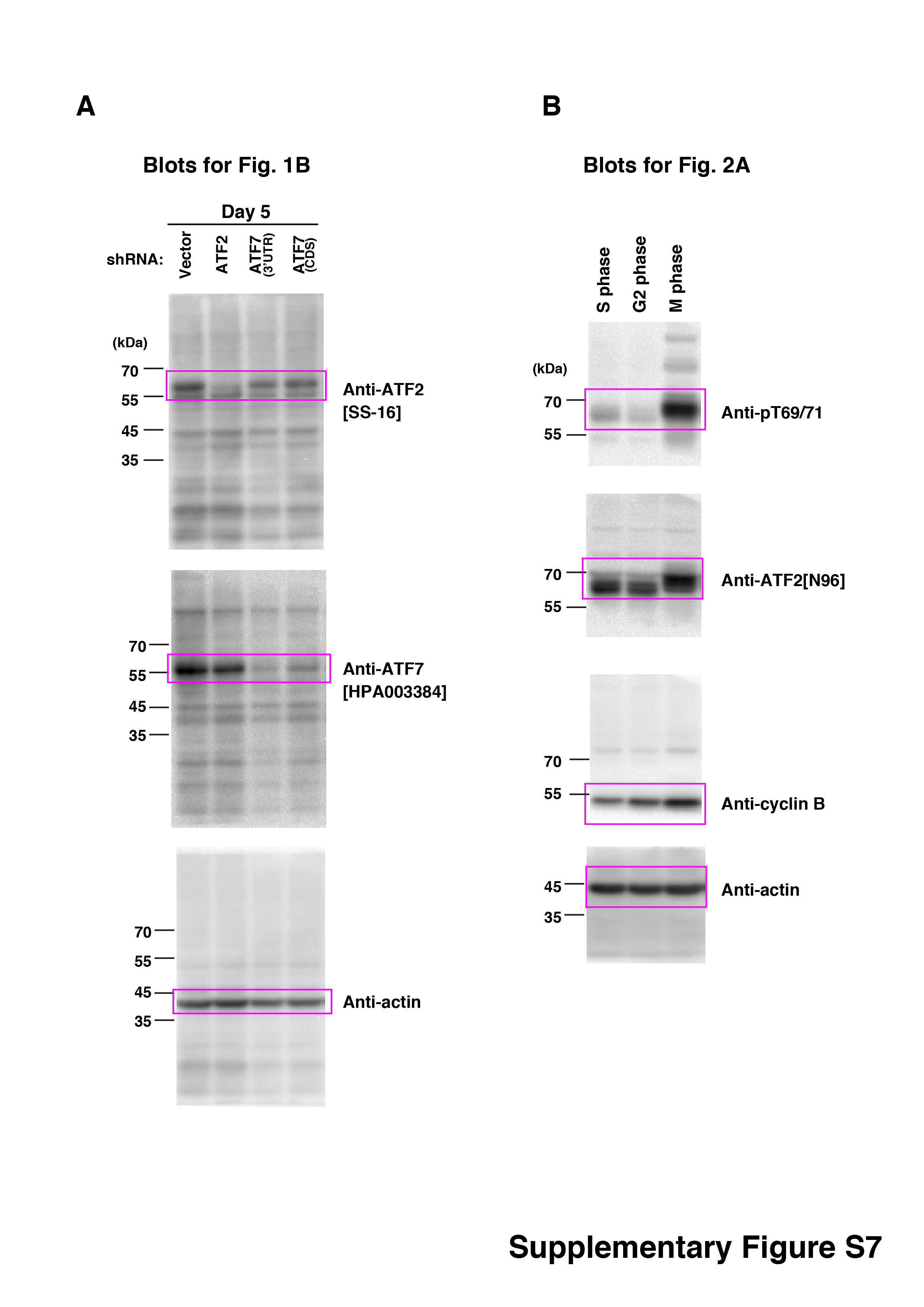

Supplement: S7 Fig — Full-length blots for Fig. 1B and Fig. 2A . (TIF) [file pone.0116048.s007.tif]

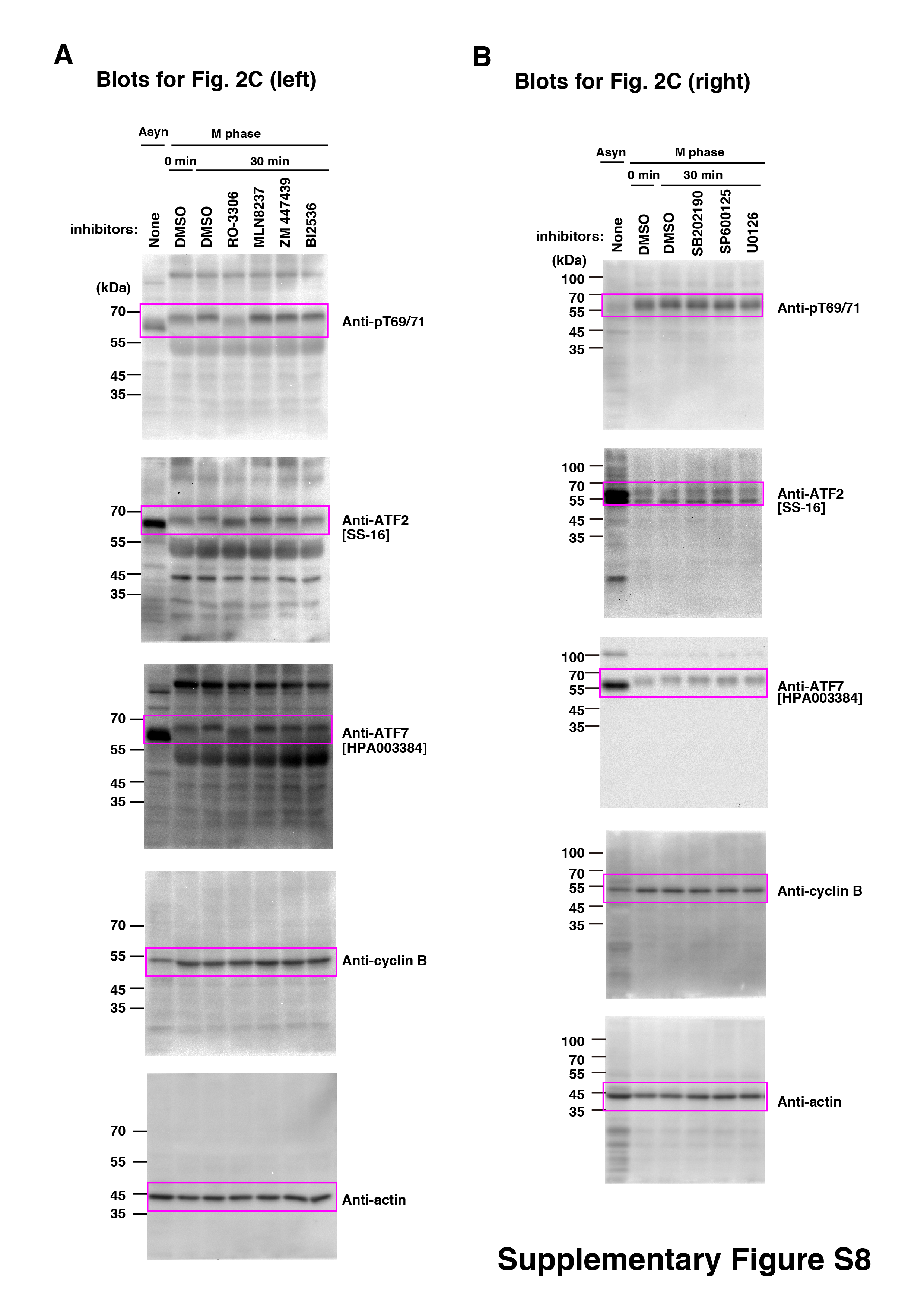

Supplement: S8 Fig — Full-length blots for Fig. 2C . (TIF) [file pone.0116048.s008.tif]

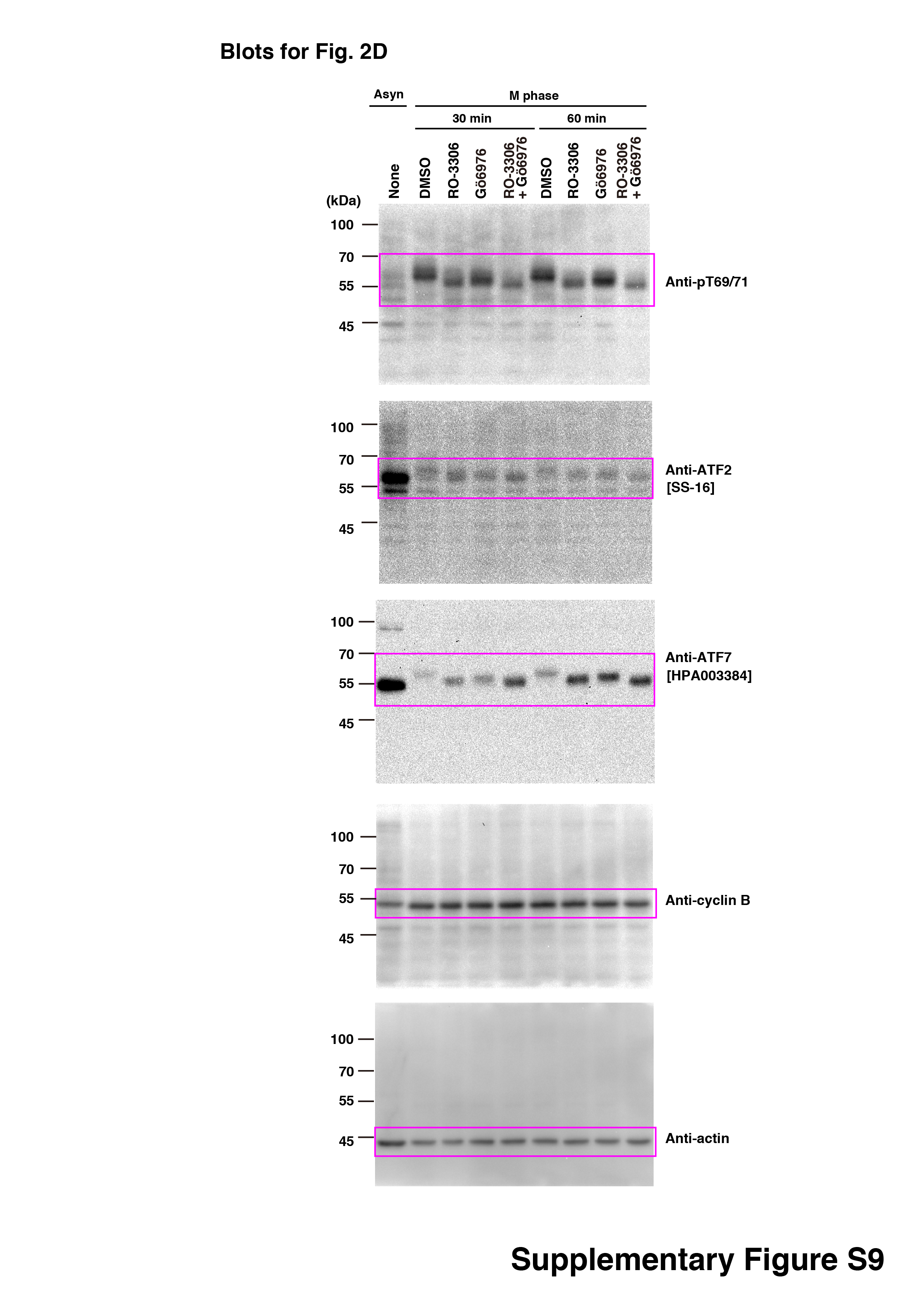

Supplement: S9 Fig — Full-length blots for Fig. 2D . (TIF) [file pone.0116048.s009.tif]

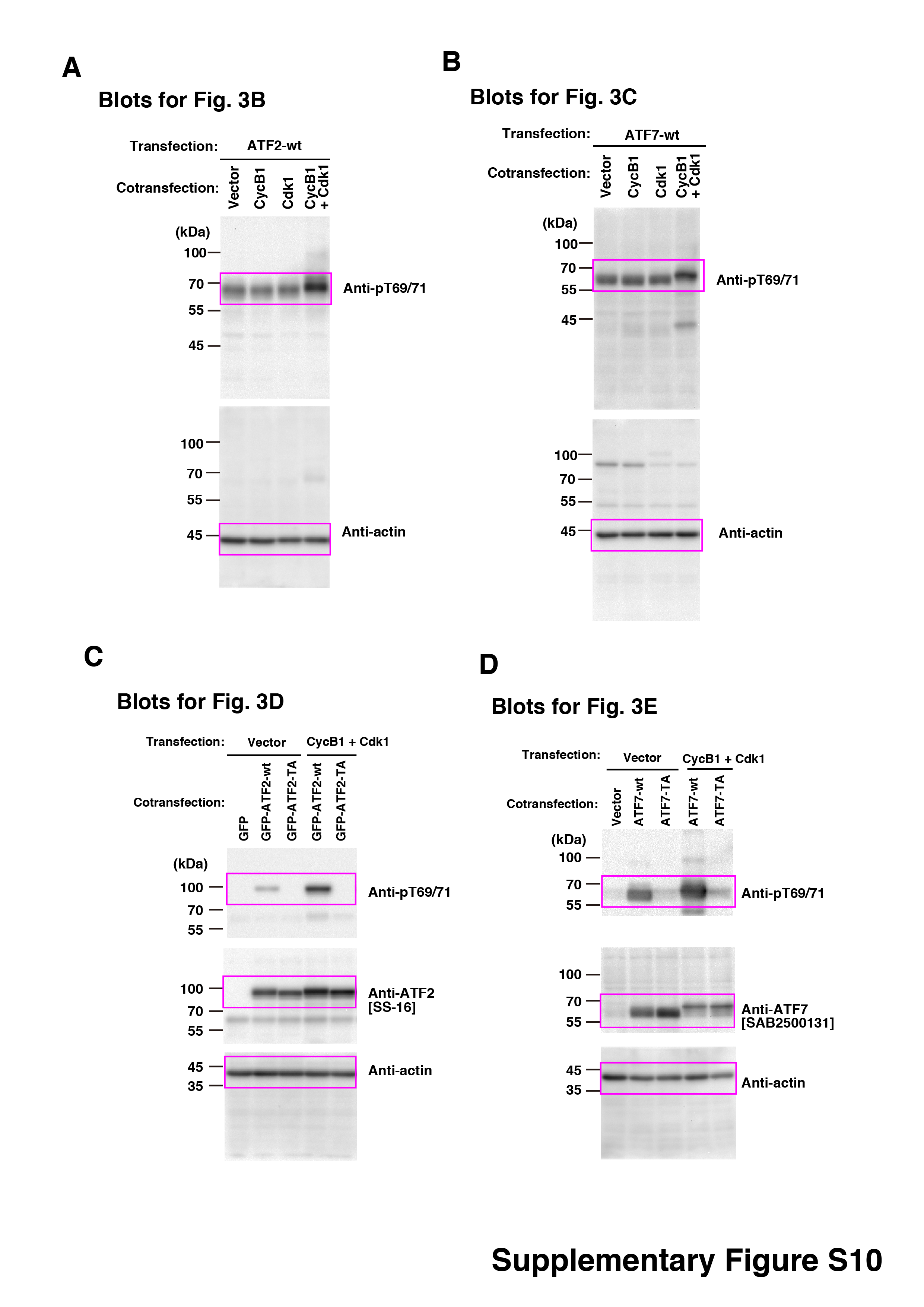

Supplement: S10 Fig — Full-length blots for Fig. 3B, 3C, 3D, and 3E . (TIF) [file pone.0116048.s010.tif]

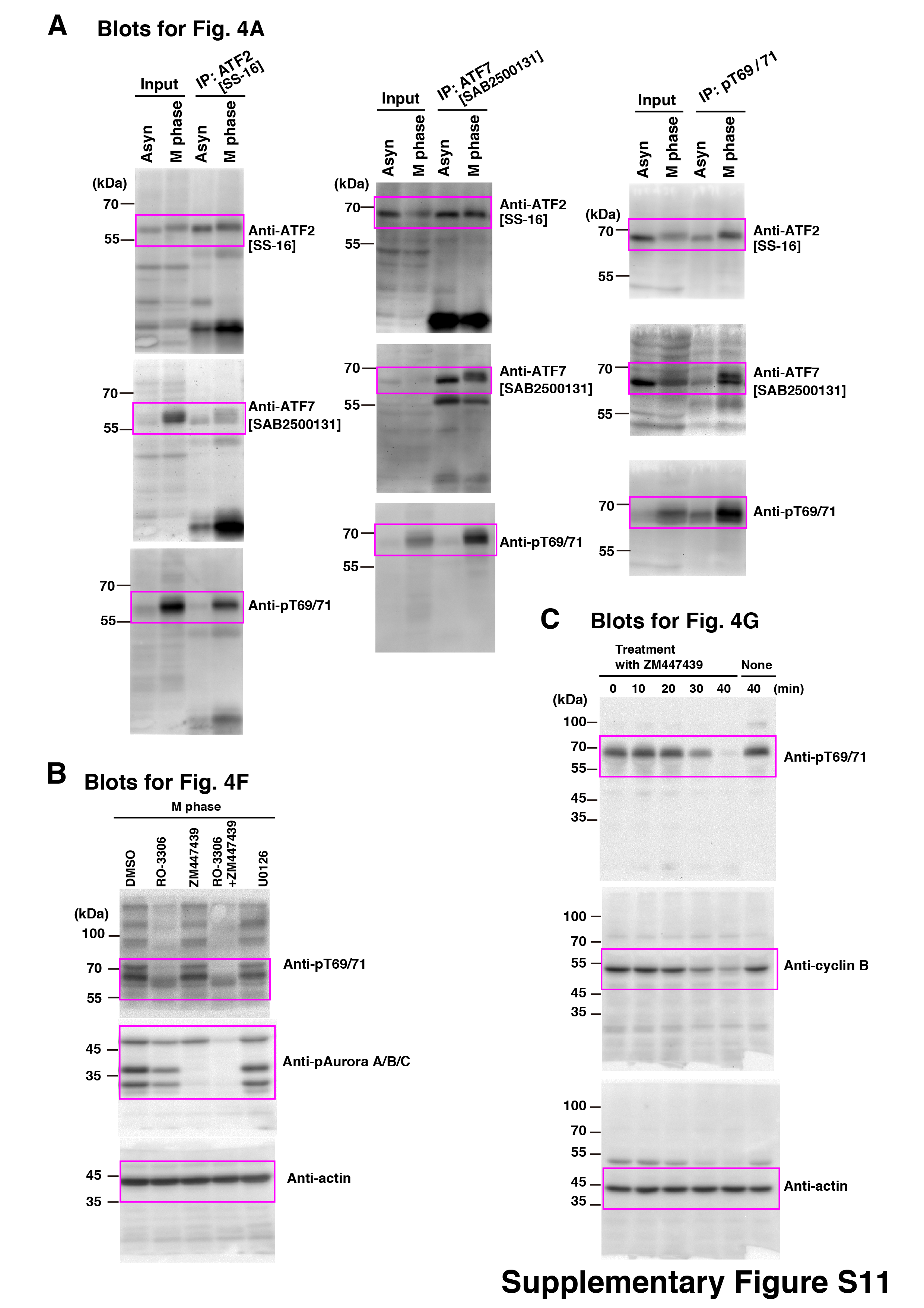

Supplement: S11 Fig — Full-length blots for Fig. 4A, 4F, and 4G . (TIF) [file pone.0116048.s011.tif]

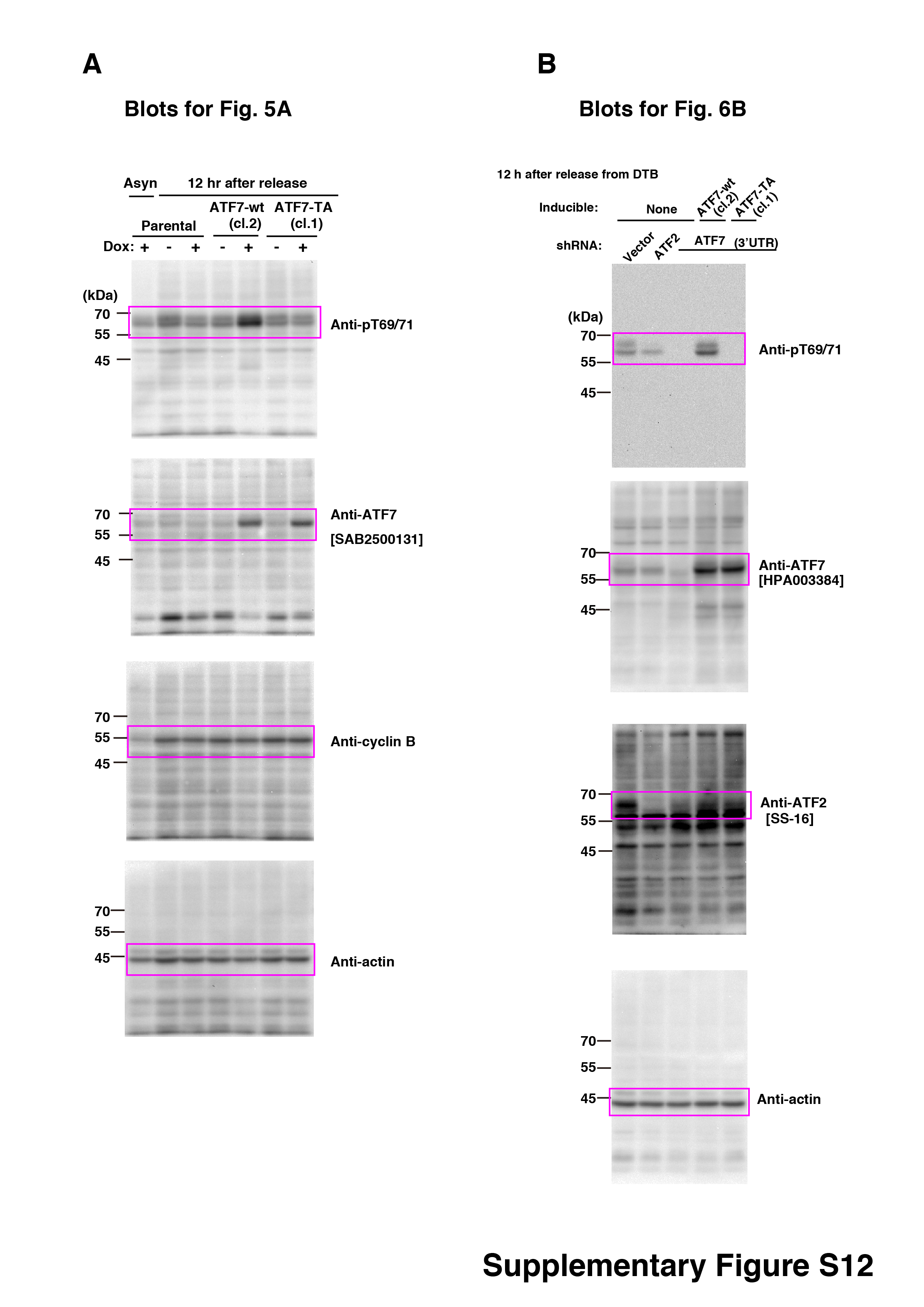

Supplement: S12 Fig — Full-length blots for Fig. 5A and 6B . (TIF) [file pone.0116048.s012.tif]

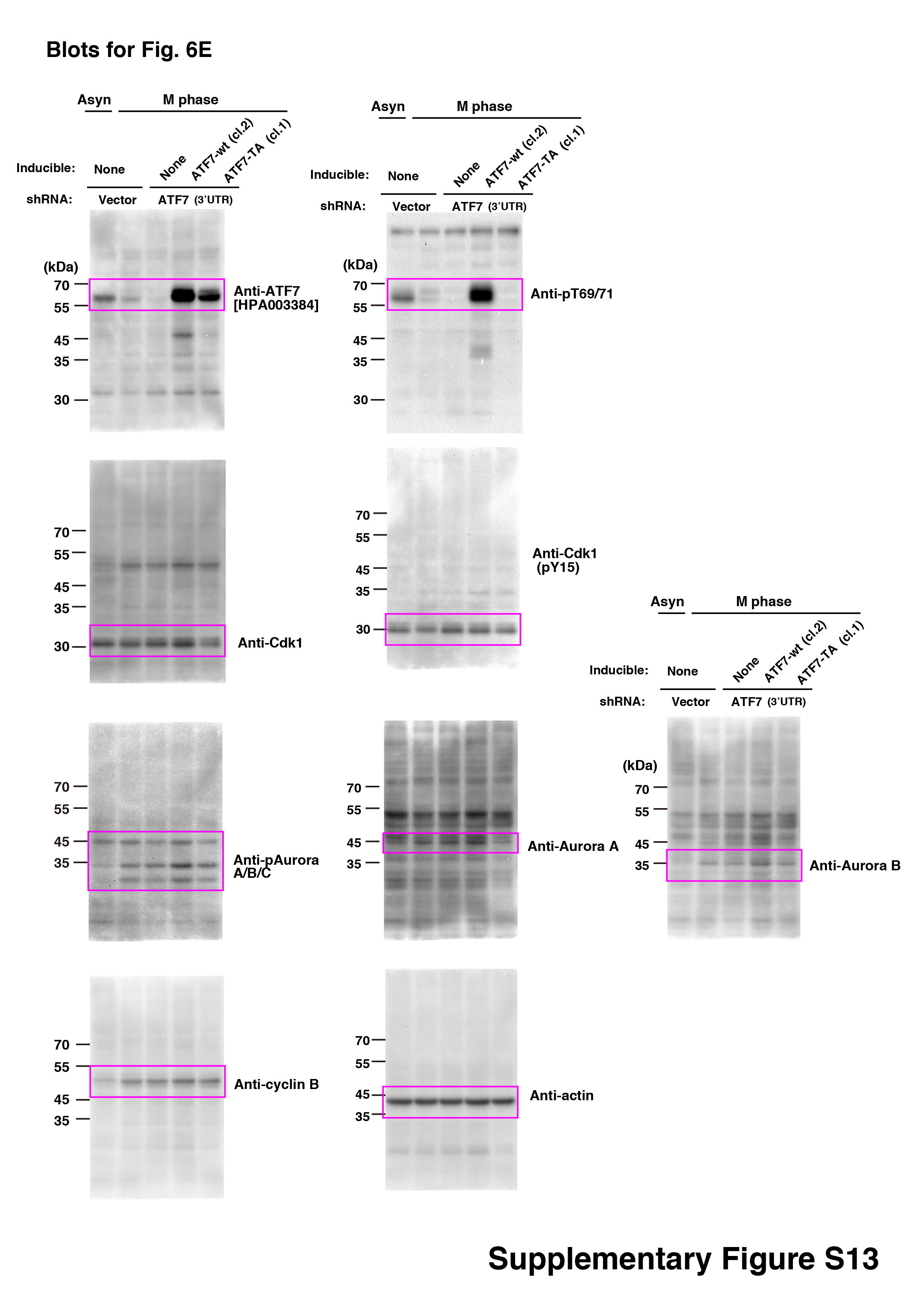

Supplement: S13 Fig — Full-length blots for Fig. 6E . (TIF) [file pone.0116048.s013.tif]

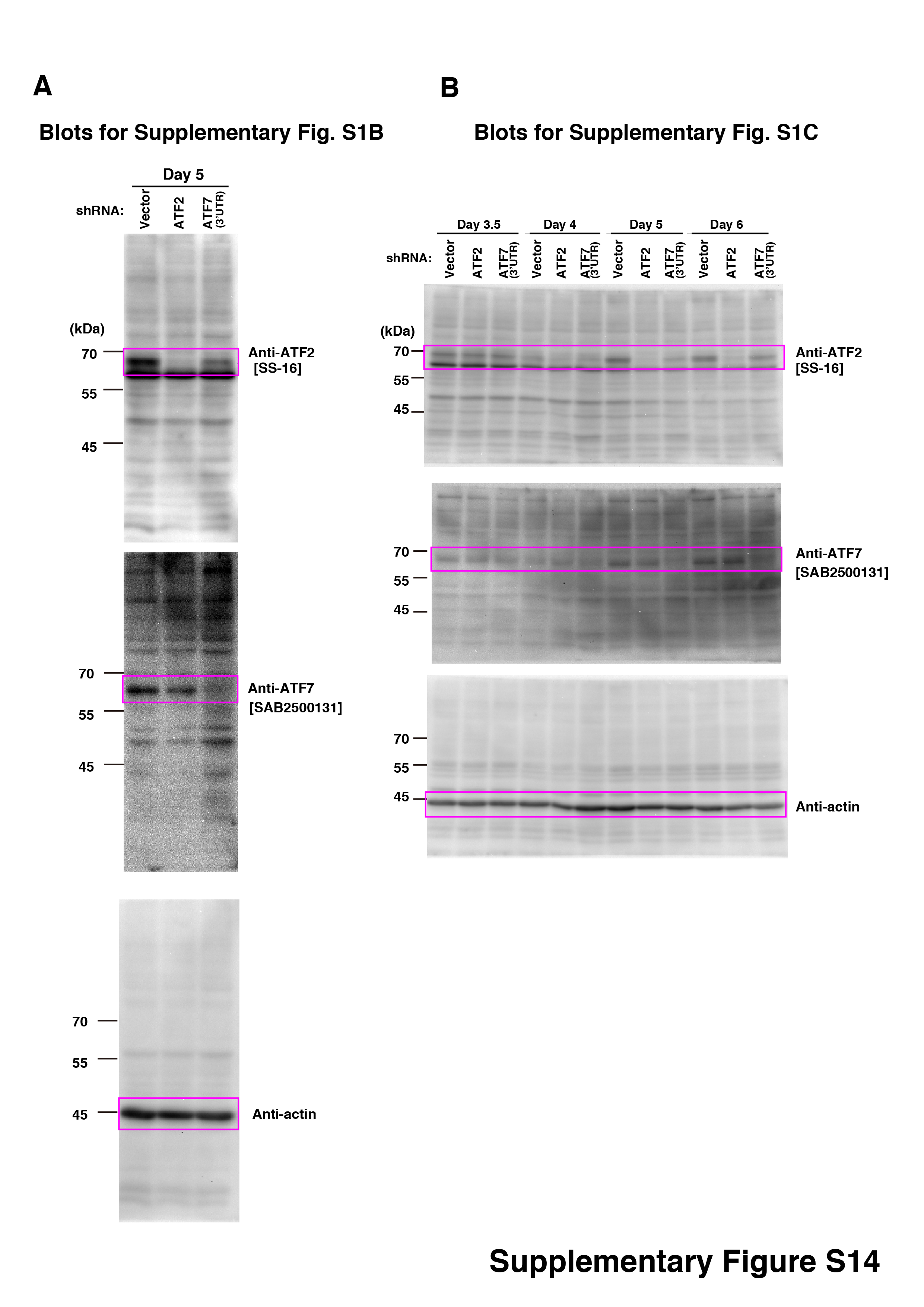

Supplement: S14 Fig — Full-length blots for S1B, C Fig. (TIF) [file pone.0116048.s014.tif]

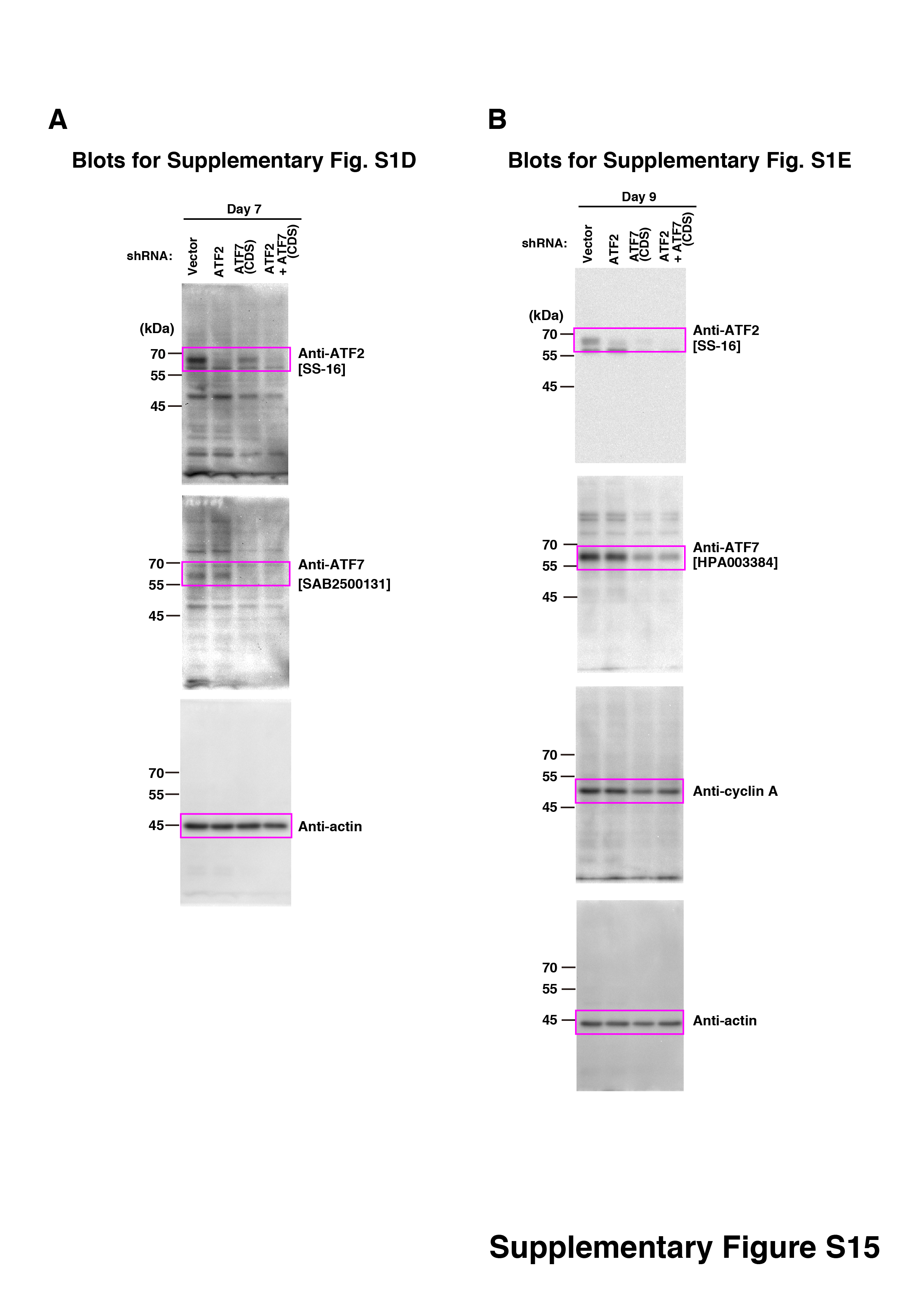

Supplement: S15 Fig — Full-length blots for S1D, E Fig. (TIF) [file pone.0116048.s015.tif]

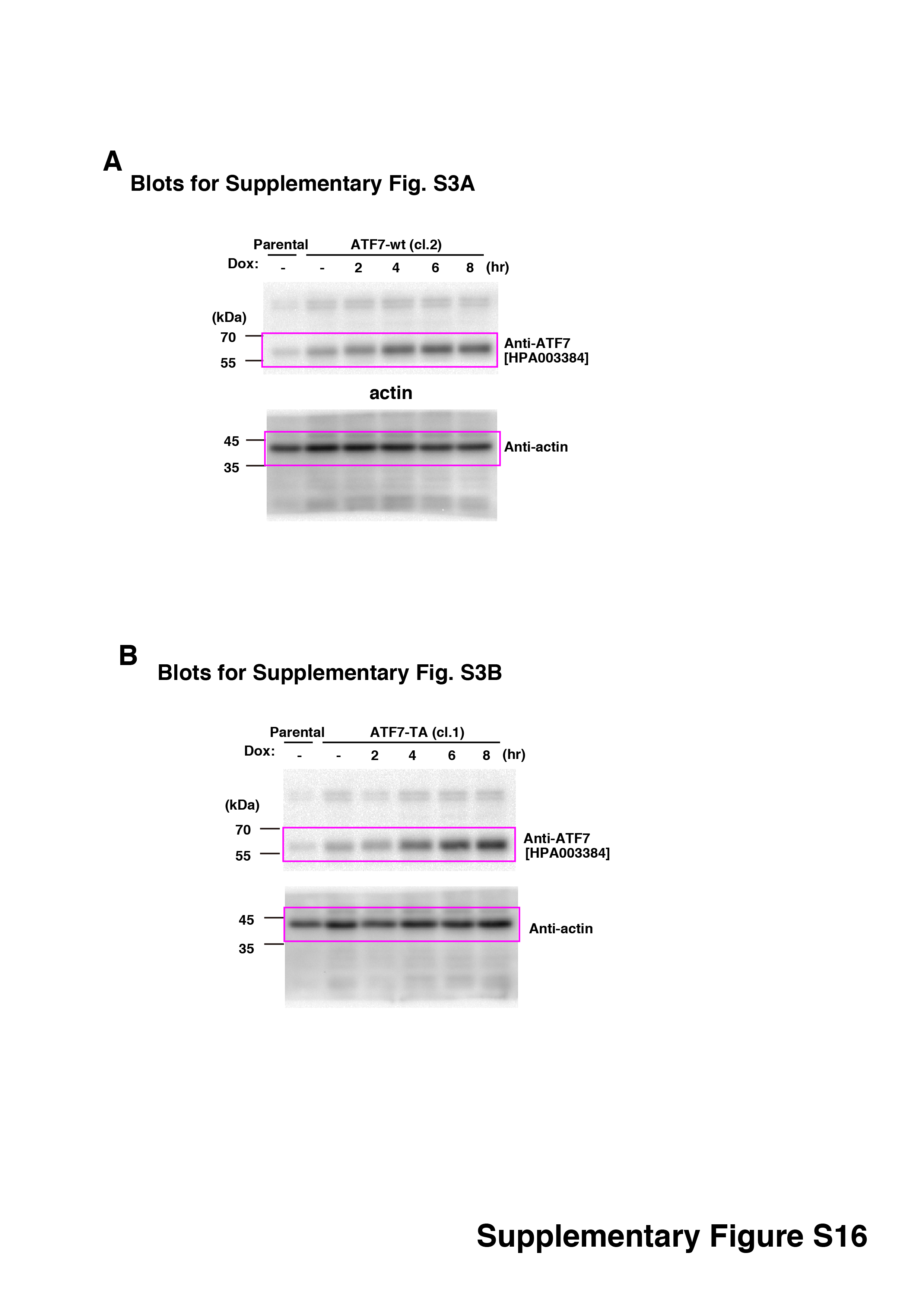

Supplement: S16 Fig — Full-length blots in S3A, B Fig. (TIF) [file pone.0116048.s016.tif]

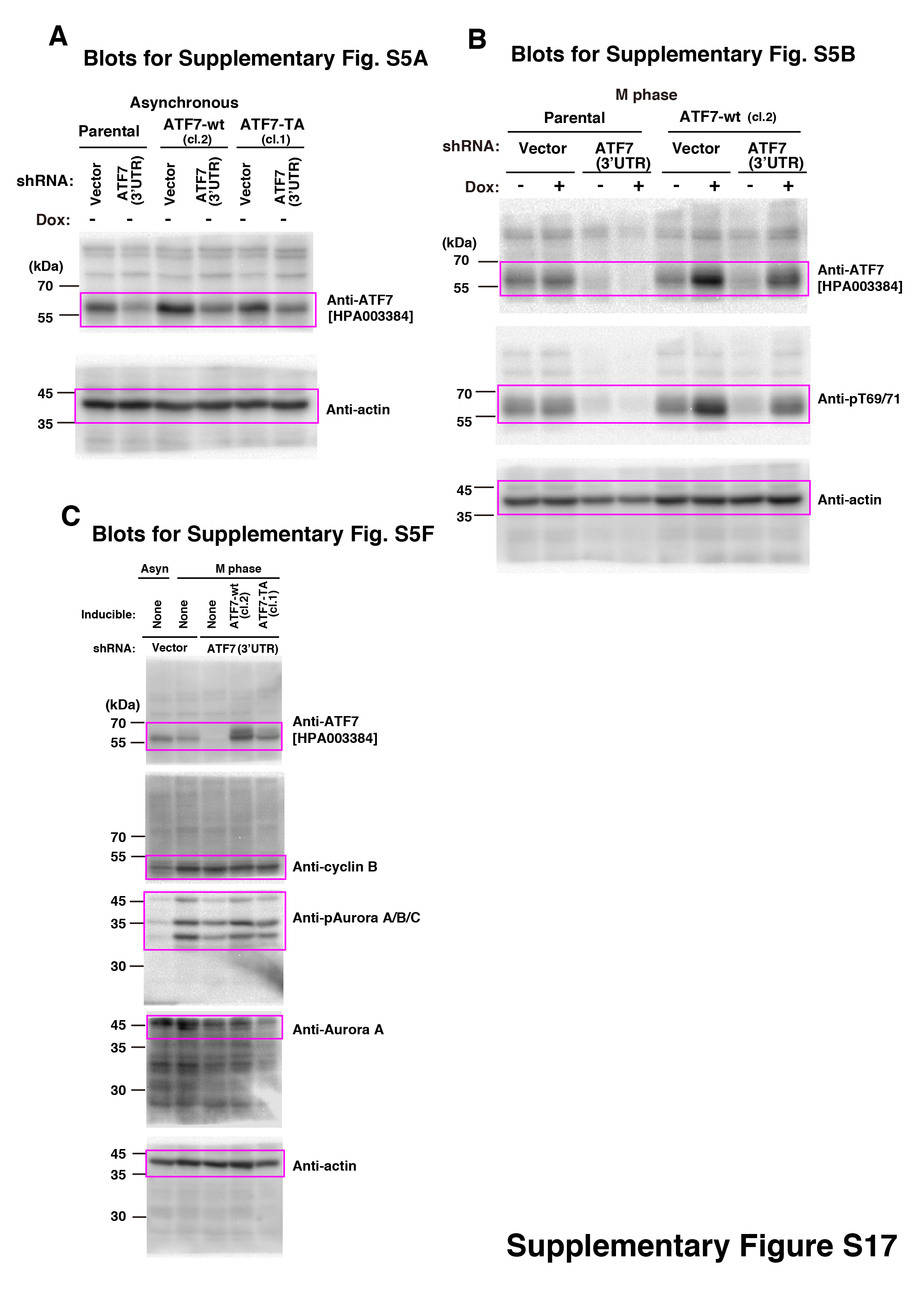

Supplement: S17 Fig — Full-length blots for S5A, B, and F Fig. (TIF) [file pone.0116048.s017.tif]

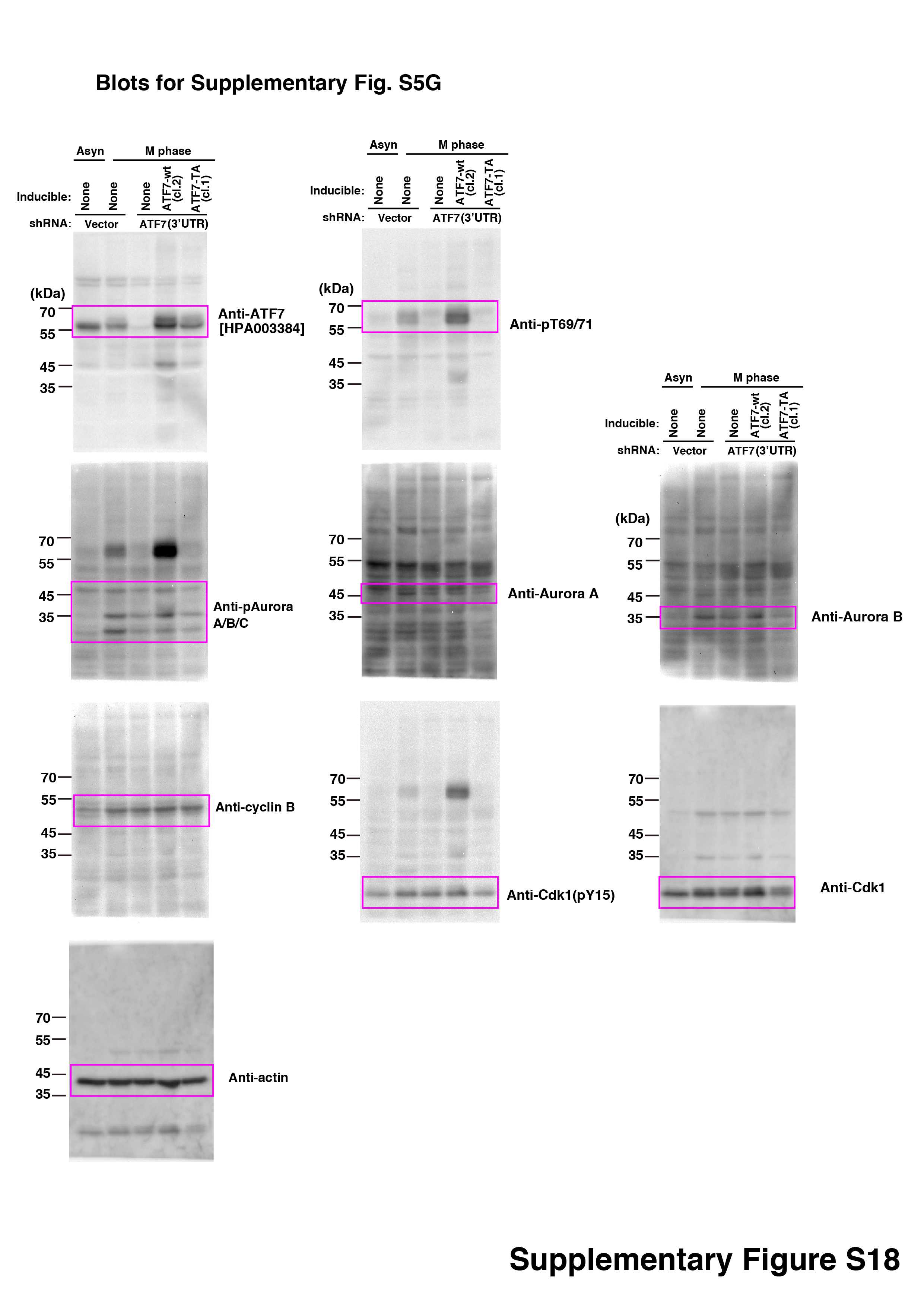

Supplement: S18 Fig — Full-length blots for S5G Fig. (TIF) [file pone.0116048.s018.tif]
